# Supplementary material for: Transformation of hard pollen into soft matter
Source: Nat Commun. 2020 Mar 19;11:1449. doi: 10.1038/s41467-020-15294-w (PMC7081183; doi:10.1038/s41467-020-15294-w)
Supplement: Supplementary file 1 — Supplementary Information [file 41467_2020_15294_MOESM1_ESM.pdf]

Transformation of Hard Pollen into Soft Matter

Fan et al.

1. Supplementary Methods

Constitutive law for intine layer based on a multiphysics model Based on the experimental results, the intine layer of pollen microgel particles is shown to behave as a stimuli-responsive hydrogel, which provides the driving force for inflation and deflation of the outer exine layer in pollen microgel particles. For simplification, the pollen intine immersed in bath solution with mobile ions are represented approximately by spherical shell structures without apertures, as shown in **Supplementary Fig. 13b**. The equilibrium of pollen intine swelling was modeled in the radial direction due to spherical symmetry. Therefore, the present computational domain consists of (i) the pollen intine layer represented by a shell-like structure, (ii) the surrounding buffer medium, including both internal and external solutions, and (iii) the interface over the pollen intine and solution domains. Since the pollen grain is an open system with three apertures, we assumed that all of the conditions of the internal solution were identical to those of the external solution.

For theoretical formulation of the model in Lagrangian scheme, several assumptions were made as follows: (i) the pollen intine system is maintained at isothermal condition, such that the dissociation constant K is independent of temperature, (ii) no chemical reaction occurs for generation of extra ions, and (iii) the pollen intine is placed in an unstirred solution and thus the effect of convection on ionic diffusion is negligible[1]. In line with the experiments, all four diffusive species in the system, including those in the pollen intine and surrounding solution, are considered in the present multiphysics model, namely hydronium ions (H_3O^+, denoted as H^+), hydroxide ions (OH^-), chloride ions (Cl^-), and respective cations (*i.e.*, K^+ or Ca^{2+}). According to the law of mass conservation, the Nernst-Planck equation is used for characterizing ionic diffusion, as described below[2],

$$\nabla_{\boldsymbol{X}} \cdot \boldsymbol{J}_k(\boldsymbol{X}) = 0, \qquad (k = \mathrm{H}^+, \mathrm{Cl}^-, \mathrm{K}^+, \text{or } \mathrm{Ca}^+) \qquad (2)$$

where $\boldsymbol{X}$ is the material coordinate and $\boldsymbol{J}_k(\boldsymbol{X})$ is the nominal flux of each mobile ion in the solution, defined as,

$$\boldsymbol{J}_k(\boldsymbol{X}) = \boldsymbol{C}^{-1}[\nabla_{\mathrm{X}} C_k + z_k C_k F_r \nabla_{\mathrm{X}} \psi / (R_0 T)] \qquad (3)$$

where z_k and C_k (mM) are the valence number and molar concentration of the ionic species k, Ψ (V) is the electric potential, and R_0, T and F_r are the universal gas constant (8.314 J $mol^{-1} \cdot K^{-1}$), the room temperature (298 K), and Faraday constant (9.6487×10^4 C mol^{-1}), respectively. In addition, $\boldsymbol{C} = \boldsymbol{F}^T \boldsymbol{F}$, where $\boldsymbol{F} = \partial \boldsymbol{x} / \partial \boldsymbol{X} = (\lambda_r, \lambda_\theta, \lambda_\varphi)$ is the deformation gradient. $\lambda_\theta = u_r / r$ is the deformation gradient in the circumferential direction, where u_r is the displacement of the spherical pollen intine in the radial direction, and, in turn, is equivalent to the swelling ratio (λ) described in this study.

For the distributive electric potential, the Poisson equation is given as follows[2],

$$\nabla_{\mathrm{X}} \cdot (J \varepsilon_0 \varepsilon_r \boldsymbol{C}^{-1} \nabla_{\mathrm{X}} \psi) = -F(z_f C_f + \sum_k C_k z_k) \qquad (4)$$

where $J = \det \boldsymbol{F}$ is the change in volume of the pollen intine layer due to swelling, ε_0 is the vacuum dielectric permittivity (8.854×10^{-12} C^2 $N^{-1} \cdot m^{-2}$), ε_r is the relative dielectric permittivity, z_f is the valence number of the fixed charge, and C_f is the fixed charge density, as given below[3],

$$C_f = \frac{C_m^0 JK}{JK + C_{\mathrm{H}^+}} \qquad (5)$$

where C_m^0 is the initial molar concentration of ionizable groups within the pollen intine in the dry state, K (mM) is the dissociation constant, and C_{H^+} is the molar concentration of hydronium ions. It is noteworthy that the fixed charge density in the multiphysics model is not constant and is varied depending on the ionic concentrations and swelling ratios.

By the law of momentum conservation, the governing equation for mechanical equilibrium with nonlinear deformation is presented as follows[4],

$$\nabla_{\mathbf{X}} \cdot \boldsymbol{P} = 0 \qquad (6)$$

where $\boldsymbol{P} = \boldsymbol{P_e} + \boldsymbol{P_{os}}$ is the nominal stress tensor contributed by two components, namely the elastic stress $\boldsymbol{P_e}$ and nominal osmotic pressure $\boldsymbol{P_{os}}$, and thus it is formulated by

$$\boldsymbol{P} = G(\boldsymbol{F} - \boldsymbol{F}^{-T}) - J\Pi\boldsymbol{F}^{-T}. \qquad (7)$$

For the effect of mechanical equilibrium on pollen intine swelling, the mechanical governing equation with nonlinear deformation is employed as[4],

$$\nabla_{\mathbf{X}}\left(G(\boldsymbol{F} - \boldsymbol{F}^{-T}) - J\Pi\boldsymbol{F}^{-T}\right) = 0 \qquad (8)$$

where G (GPa) is the pollen intine shear modulus associated with the Young's modulus E and the Poisson's ratio υ via $\mathrm{G} = \mathrm{E}/[2(1+\upsilon)]$, and Π is the osmotic pressure given by[5],

$$\Pi = -\frac{k_B T}{v_s}\left(\ln(\frac{v_s C_s}{1+v_s C_s}) + \frac{1}{1+v_s C_s} + \chi_H(\frac{1}{1+v_s C_s})^2\right) + k_B T\left(\frac{1}{C_s v_s}\sum_k C_k - \sum_k \overline{C}_k\right) \qquad (9)$$

where the overbar ($\overline{C_k}$) denotes the ion concentration in the solution, $k_B T$ is the temperature in the unit of energy, v_s is the volume per solvent species, C_s is the number of solvent species divided by the volume of the dry polymer, and χ_H is the Flory-Huggins parameter to characterize the interactions between solvent molecules and the polymer network inside the pollen intine. From Equation (6), it is found that the osmotic pressure inside the pollen intine Π is due to mixing of the polymer and solvent molecules, as well as due to the imbalance of ions in the external solution and the pollen intine, including mobile ions and fixed charges.

The swelling-induced Cauchy stress is given by

$$\boldsymbol{\sigma} = (\sigma_r, \sigma_\theta, \sigma_\varphi) = \frac{1}{J} G(\boldsymbol{F}\boldsymbol{F}^T - \boldsymbol{I}) - \Pi\boldsymbol{I} \qquad (10)$$

where $\boldsymbol{I}$ is a unit matrix and $\sigma_\theta = \sigma_\varphi$ for a spherical symmetric system.

The stress in the radial direction is then obtained by substituting the deformation gradient and osmotic pressure into Equation (7), as follows:

$$\sigma_r = \frac{1}{J}G(\lambda_r{}^2 - 1) + \frac{k_B T}{v_s}\left(\ln(\frac{v_s C_s}{1+v_s C_s}) + \frac{1}{1+v_s C_s} + \chi_H (\frac{1}{1+v_s C_s})^2 \right) - k_B T\left(\frac{1}{C_s v_s}\sum_k C_k - \sum_k \bar{C}_k \right) \quad (11)$$

As shown in **Supplementary Fig. 13b**, for the spherical symmetric problem, the Neumann type of the electrochemical boundary conditions are imposed at the center of the pollen, located at $\boldsymbol{X} = 0$, namely,

$$\frac{\partial C_k}{\partial \boldsymbol{X}} = 0,\ \frac{\partial \psi}{\partial \boldsymbol{X}} = 0 \qquad (12)$$

and Dirichlet boundary conditions are imposed at the edge of the solution domain,

$$C_k = \bar{C}_k,\ \psi = 0 \qquad (13)$$

A free swelling process is conducted to identify the ionic concentrations at equilibrium, and an equilibrium state over the spherical shell-solution interface is given as

$$G(\boldsymbol{F} - \boldsymbol{F}^{-T}) - J\Pi\boldsymbol{F}^{-T} = 0 \qquad (14)$$

The driving pressure is then achieved by inputting the equilibrium ionic concentrations into Equation (8). The simulation was conducted by the COMSOL Multiphysics 5.3 software package (COMSOL Inc., Stockholm, Sweden). The partial differential equation (PDE) interfaces were used for capturing ionic transport and pollen intine deformation, and the electrostatics module was used to describe the electric distribution in the domain. All the dependent variables in the PDEs and electrostatics module were computed by the fully coupled solver. In order to perform numerical simulations of pollen intine swelling in the ionic solution, all the inputs required by the multiphysics model are tabulated in **Supplementary Table 2**. Among the inputs required in the present model, only the initial fixed charge density in the dry state C_m^0 was not directly obtained in the experiment. Thus, by an approach to the inverse problem, $C_m^0 = 1.7848 \times 10^4$ mM was identified through the present model associated with the experimental data, where the swelling of the deformed intine layer ($E = 250$ MPa) was $\lambda = 1.5946$ at pH = 7. Moreover, the constant $C_m^0 = 1.7848 \times 10^4$ mM was further validated with the FEA modeling of the exine layer where intine swelling was constrained by the external exine layer, thus free swelling of the intine layer became swelling-induced mechanical pressure exerted to the internal surface of the exine layer. Since C_m^0 is an initial material property of the pollen intine, and is independent of the pollen intine swelling process, the well-identified value of C_m^0 could provide consistent modeling prediction which showed good agreement with experimental observations.

Constitutive law for exine layer The maximum swelling diameter of pollen microgel particles reached up to 1.8 times the size of their original diameter, as seen for the case of 6 h KOH-treated pollen samples. Large deformation around the tips of the three apertures can occur due to the high-stress concentration factor at the tip of a crack[6]. Moreover, the swelling/de-swelling behavior of pollen microgel particles was reversible. Thus, the particles clearly showed rubber-like hyperelastic behavior. Therefore, we built three-dimensional computational models for simulating the micromechanics of the exine layer in pollen particles based on the classic hyperelastic neo-Hookean model[7]. In the context of pollen microgel particles, about the paucity of reliable data on material properties of exine layer and the lack of detailed studies of its

constitutive response led us to choose the simple but efficient neo-Hookean model, rather than complex models with many adjustable parameters.

The strain energy density of the neo-Hookean model is given by

$$\mathrm{U} = C_{10}\left(\left(J^{-\frac{1}{3}}\lambda_1\right)^2 + \left(J^{-\frac{1}{3}}\lambda_2\right)^2 + \left(J^{-\frac{1}{3}}\lambda_3\right)^2 - 3\right) + \frac{1}{D_1}(J-1)^2 \qquad (15)$$

where U is the elastic strain energy per unit reference volume; λ_1, λ_2, and λ_3 are the three principal stretches; and J is the total volume ratio, defined as $\mathrm{J} = \lambda_1\lambda_2\lambda_3$[7,8]. Two material constants, C_{10} and D_1, were chosen to simulate the inflation and deflation behavior of the exine layer under large elastic strain. C_{10} is related to the initial shear modulus G as follows,

$$C_{10} = \frac{1}{2}G \qquad (16)$$

$$\mathrm{D}_1 = \frac{2}{\mathrm{K_b}} \qquad (17)$$

where $\mathrm{K_b}$ is the bulk modulus, and G and $\mathrm{K_b}$ are related to the Young's modulus E by the formulas, $\mathrm{G} = \mathrm{E}/2(1+\upsilon)$ and $K_b = \mathrm{E}/3(1-2\upsilon)$ with the Poisson's ratio, υ. The Young's modulus was obtained from AFM force-based nanoindentation tests that were performed on the pollen particles (**Supplementary Figs. 11-12**).

G and K_b are related to the Young's modulus E, which retrieved from AFM force-based nanoindentation tests performed on the pollen particles (**Supplementary Figs. 11-12**), by the formulas, $\mathrm{G} = \mathrm{E}/2(1+\upsilon)$ and $K_b = \mathrm{E}/3(1-2\upsilon)$ with a Poisson's ratio, υ. Thus, we can express the two material constants, C_{10} and D_1, in terms of exine material parameters, E and υ, as follows,

$$C_{10} = \frac{E}{4(1+\upsilon)} \qquad (18)$$

$$D_1 = \frac{6(1-2\upsilon)}{E} \qquad (19)$$

Considering the presence of pores in the exine layer, a value of 0.4 for the Poisson's ratio was selected to account for exine compressibility.

FEA modeling for inflation/deflation of exine layer The geometry of pollen microgel particles was determined by direct experimental measurements using a scanning electron microscope (SEM). The average thickness of the exine layer was determined to be 0.58 μm, and the mean outer diameter of the particles was 28 μm. Spikes associated with the exine layer were defined as cones with a height of 5 μm and a base diameter of 3 μm. The aperture length was described relative to a central angle of 90°, and the tip radius of curvature of the aperture was set as 1.0 μm. This latter value was selected in order to avoid convergence issues related to large deformation around the aperture during finite element simulations of inflation of pollen exine (**Supplementary Fig. 13c**). A one-third symmetry structure with only one aperture and 38 spikes was constructed as the exine for one microgel particle, where symmetric boundary conditions were applied at the perimeter (**Supplementary Fig. 13d**). Furthermore, the density of exine was taken as 1 g cm^{-3}. Considering the small size and low density of the pollen grains in solvents, gravitational effects were not considered in the model. The outer surface of the exine layer was tightly attached to the

spikes, and an inflation pressure ($P_{e,inflation}$) generated by osmotic pressure of the intine ($P_{i,swelling}$) was exerted on the inner surface of the exine layer. The effects of mesh size on the deformation capacity were studied by an adaptive mesh strategy, where the largest mesh size was 0.5 μm. Therefore, various mesh sizes were employed for small deformation at regions distant from the aperture and were decreased to a minimum value of 0.02 μm for large deformation ($\varepsilon > 2$) around the tip of the aperture. Meanwhile, the artificial strain energy of the whole model associated with hourglass was less than 2% of the total internal energy. It should be noted that a severely inhomogeneous deformation was observed during the swelling process so the swelling diameter was measured by a red circle in the direction of maximum deformation, as indicated in **Supplementary Fig. 13e**. To investigate the inflation/deflation processes as well as related deformation mechanisms, we employed a three-dimensional model of the exine layer based on finite element analysis (FEA) using a commercial software package ABAQUS 2017® (Dassault Systèmes SIMULIA, Johnston, RI). The exine model was generated by Python scripts and then ran in parallel in 32 cores. Quasi-static solutions were calculated using ABAQUS/Explicit[8,9] to simulate the inflation process of the pollen exine layer with different mechanical properties under an internal pressure while kinematic effects were ignored. The finite element mesh entailed solid eight-node brick elements with reduced integration (C3D8R), while the spike on the pollen exine surface was treated as a rigid surface without deformation throughout the entire simulation. A total of 75,938 elements were used in the pollen exine model. A pseudo step time of 30 μs for inflating and 30 μs for deflating the exine structure was set in order to minimize the kinetic energy. The inflation pressure ($P_{e,inflation} = p_{int}$) was predicted by the intine swelling model ($P_{i,swelling} = \sigma_{rr}$) based on the relationship, $P_{e,inflation} = P_{i,swelling}$; thus, the boundary condition applied to the internal surface of the exine layer was $\sigma_{rr} = p_{int}$. A maximum internal pressure ($p_{int,max}$) of 8.3 MPa, induced by the swollen intine layer, was predicted by the multiphysics model that corresponded to 6 h KOH-treated pollen microgels incubated in pH 10 solution; this was the treatment condition which exhibited maximum swelling among all pollen microgel particle specimens. The reference pressure was, therefore, set as 8.3 MPa ($p_{int,max}$) and 0 MPa ($p_{int,min}$) for conditions corresponding to pH 10 and pH 2 conditions, respectively, for all of the simulations, and the Young's modulus values and the predicted internal pressure values were systematically varied in the parametric studies, as indicated in **Supplementary Table 1**. The pressure boundary condition on the inner surface of the exine layer was applied with a defined load profile gradually increasing up to 8.3 MPa and then decreasing to zero to improve the convergence of the model in large deformation simulations.

2. Supplementary Figures (1-30)

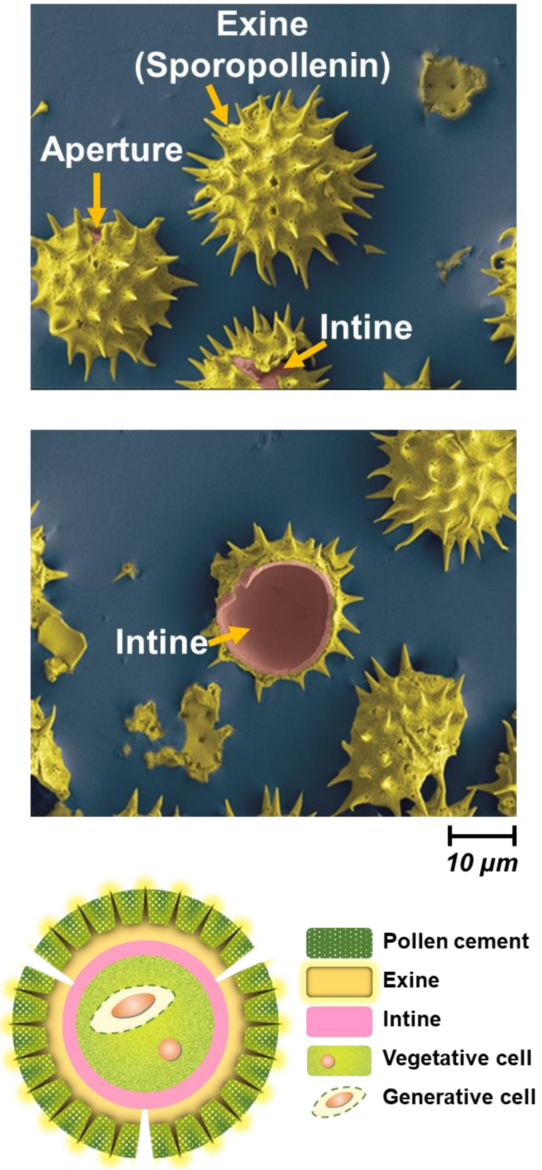

Supplementary Figure 1 | Scanning electron microscope (SEM) images and schematic illustration of sunflower pollen grains. a-b, SEM images show distinctive features of pollen grains, including the exine, apertures, and intine. The pollen structures are pseudo-colored. **c,** Schematic illustration of pollen cement, exine, intine, and genetic material.

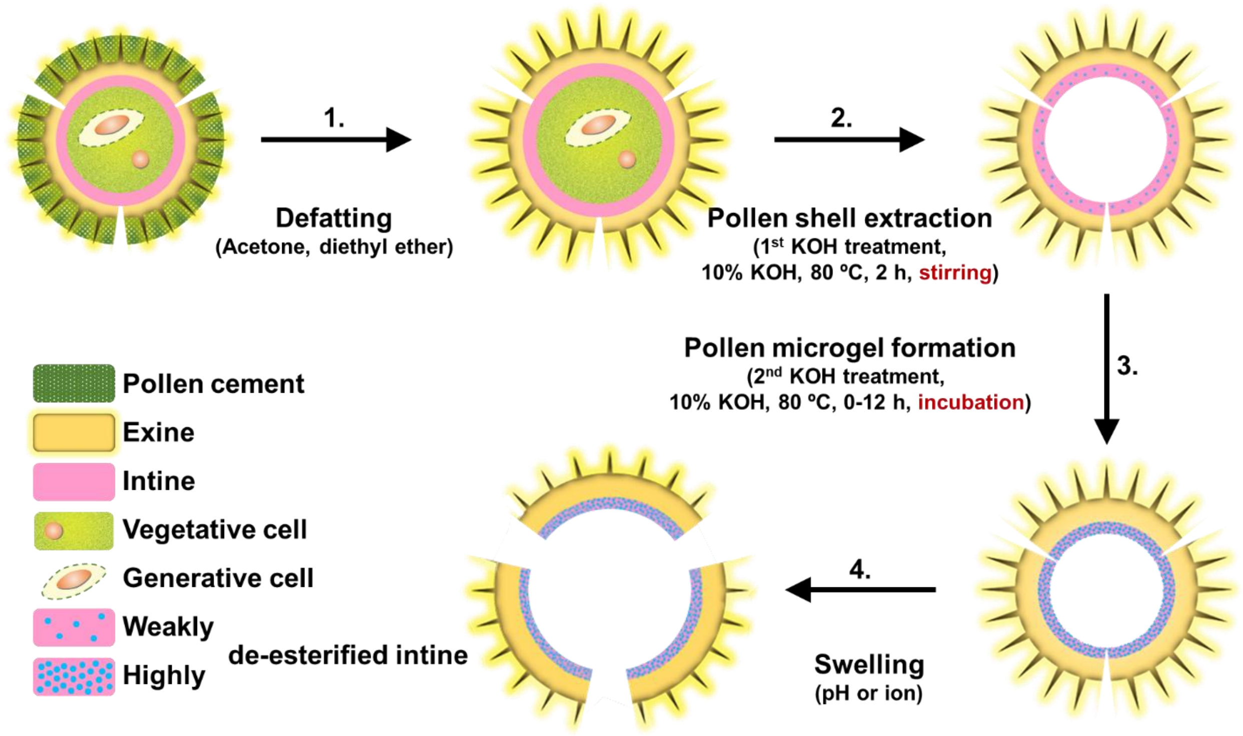

Supplementary Figure 2 | Schematic illustration of pollen microgel fabrication process. Microgel fabrication process involves the following four processing steps (as indicated by arrows): (1) Treatment with organic solvent to remove excess lipid coating; (2) Pollen incubation in alkaline conditions for 2 h at 80 °C under stirring condition ("Pollen shell extraction"); (3) Extended incubation in alkaline conditions at 80 °C for up to 12 h ("Pollen microgel formation"); and (4) Gelation of pollen microgels induced by water rinsing.

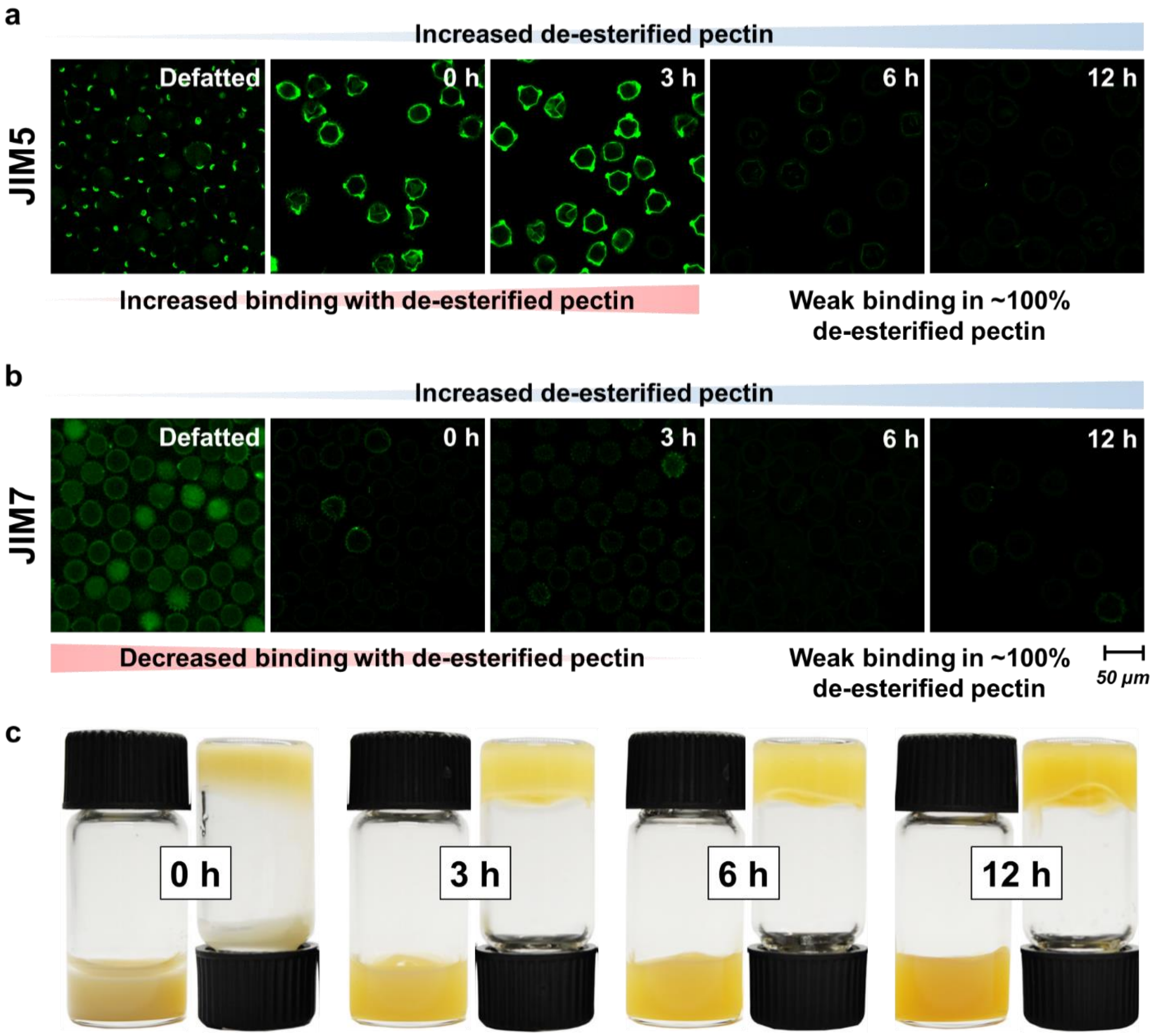

Supplementary Figure 3 | Immunofluorescence microscopy used for the detection of de-esterified pectin within pollen shells. Prior to experiments, the pollen microgel samples were incubated with **a,** JIM5 and **b,** JIM7 monoclonal antibody, which recognize weakly and highly esterified pectin molecules, respectively, and both antibodies cannot detect fully de-esterified pectin samples[10].

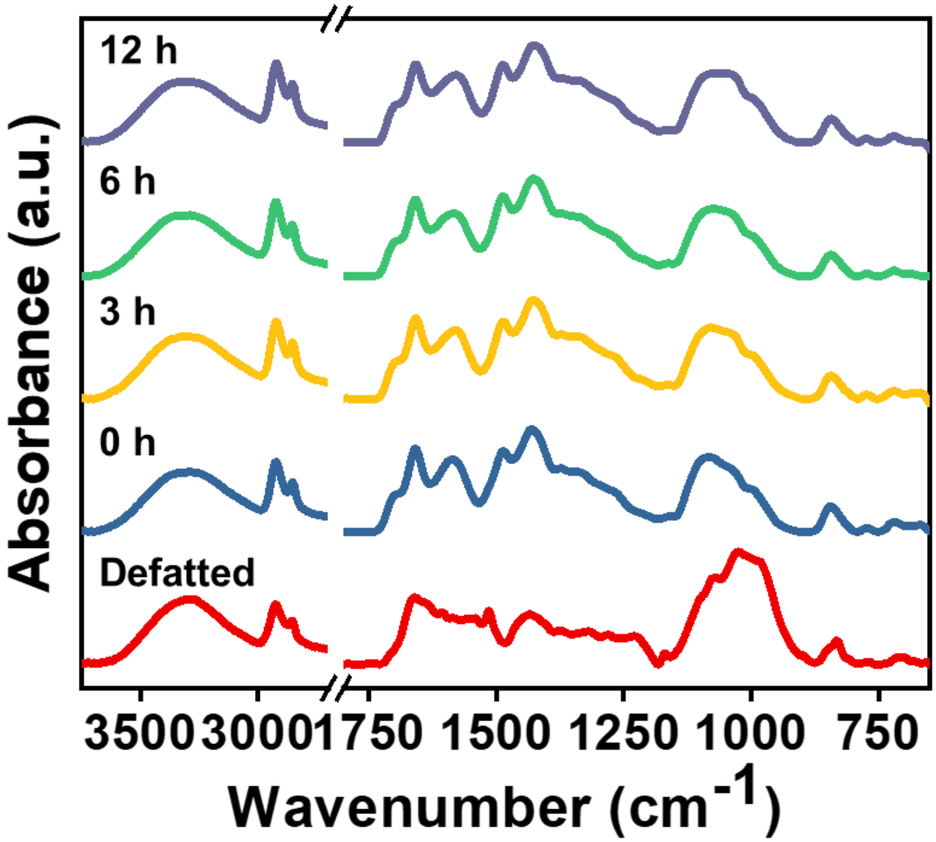

Supplementary Figure 4 | FTIR characterization of 10% (wt/vol) KOH-treated defatted sunflower pollen grains. The FTIR spectra of pollen microgel dispersions as a function of incubation time in 10% (wt/vol) KOH are presented. Pectin peaks (~1620 cm^{-1}) were more clearly observed for KOH-treated pollen grains than defatted pollen. The characteristic absorbance peaks of all KOH-treated pollen grains appear almost identical, irrespectively of the treatment time with strong alkali. Source data are provided as a Source Data file.

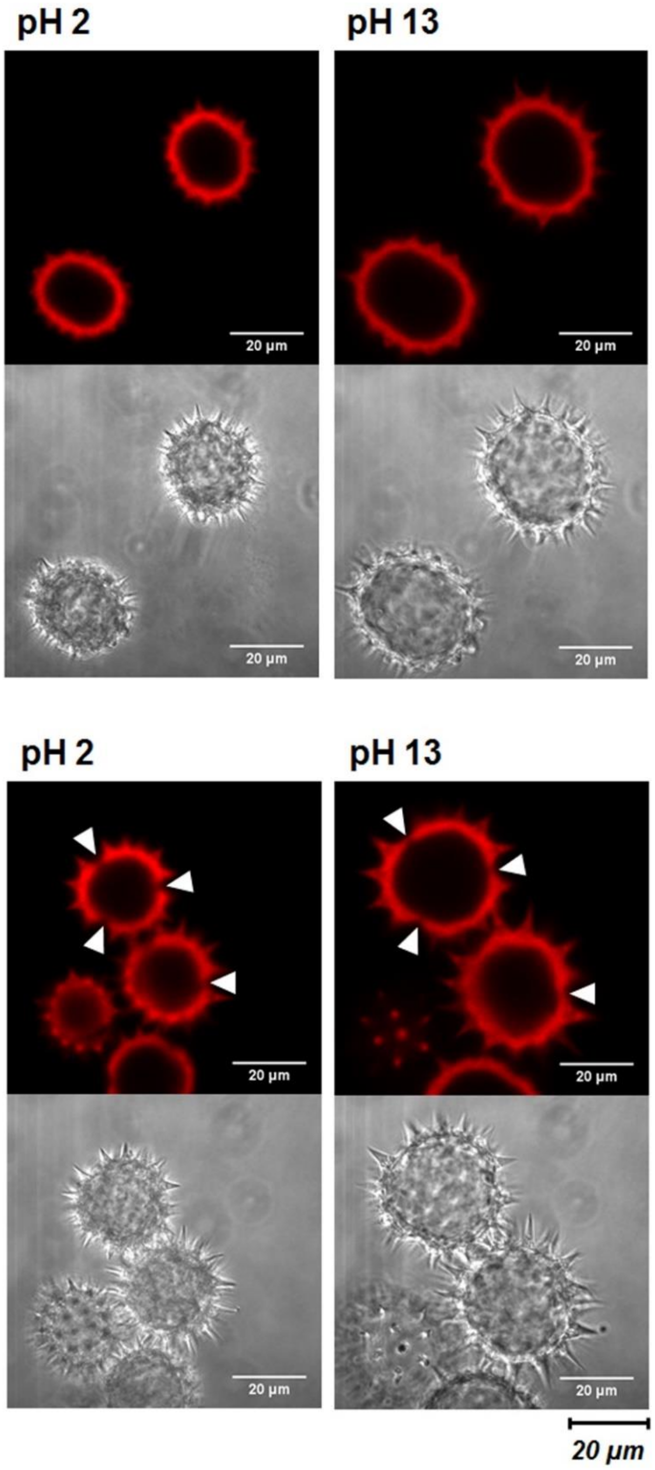

Supplementary Figure 5 | Microscopic characterization of 6 h KOH-treated defatted sunflower pollen microgel particles under different pH conditions. Cross-sectional CLSM images of pollen microgel particles in pH 2 and pH 13 conditions. The white arrows indicate the aperture openings on the pollen microgel particles. The corresponding bottom images were obtained in differential interference contrast (DIC) mode.

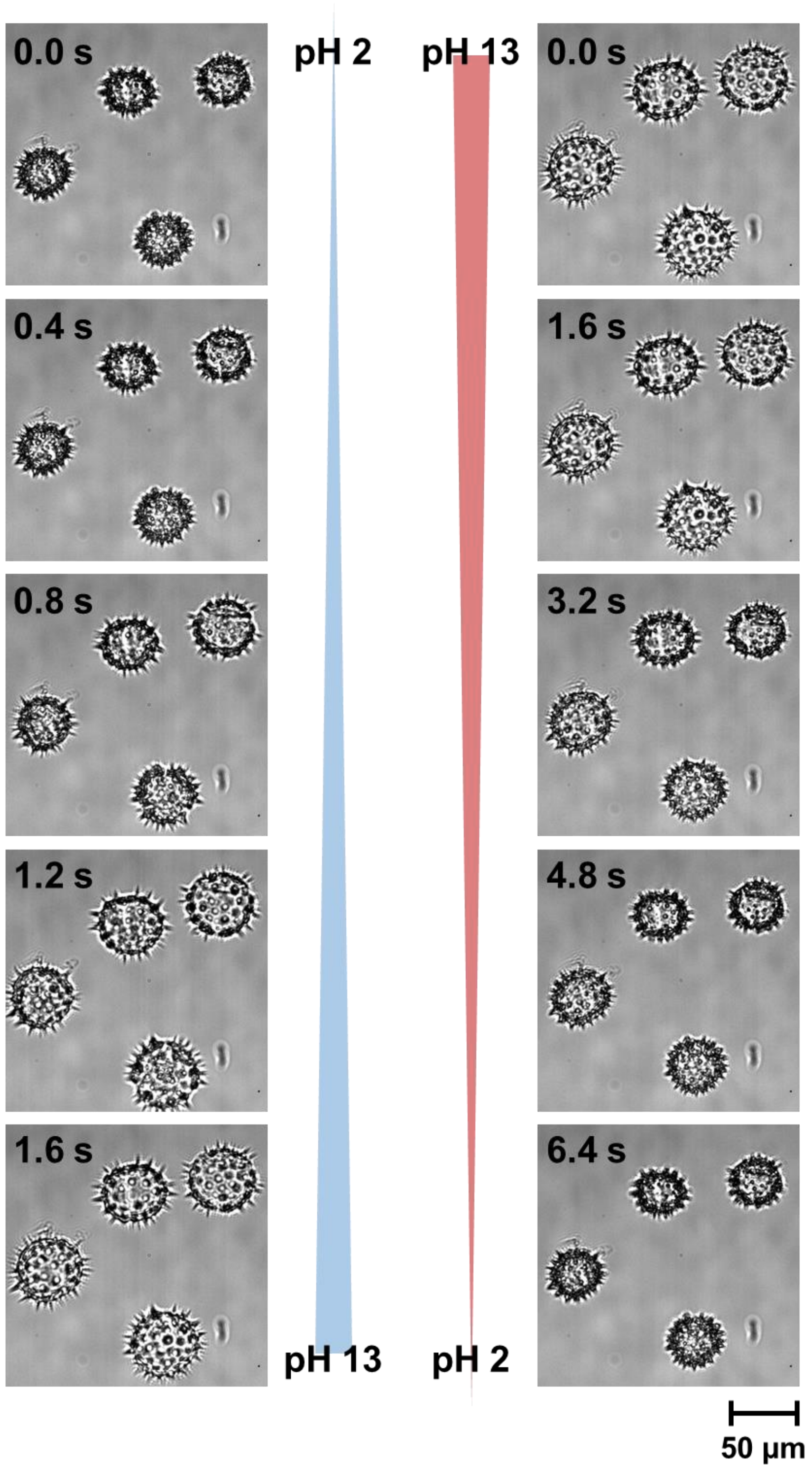

Supplementary Figure 6 | Time-lapse micrographs of 6 h KOH-treated defatted sunflower pollen particles in response to change in solution pH. Pollen microgel particles are responsive to the change of pH over a relatively short time.

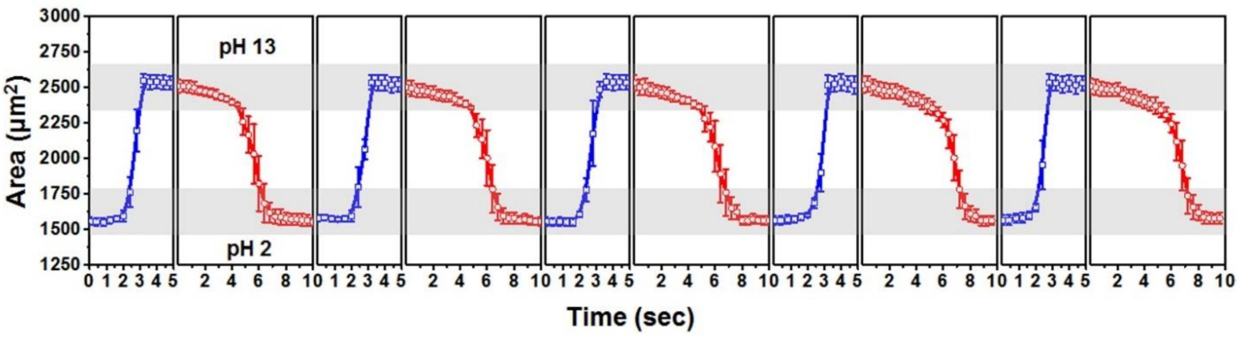

Supplementary Figure 7 | Reversible swelling and de-swelling behavior of pH-responsive pollen microgels. Repetitive gradual changes in the solution pH back and forth from pH 2 to pH 13 are presented. Time-lapse micrographs are taken in 0.4-s time intervals in order to track the area change of individual pollen microgel particles (n = 3). Data are presented as mean ± s.d. Source data are provided as a Source Data file.

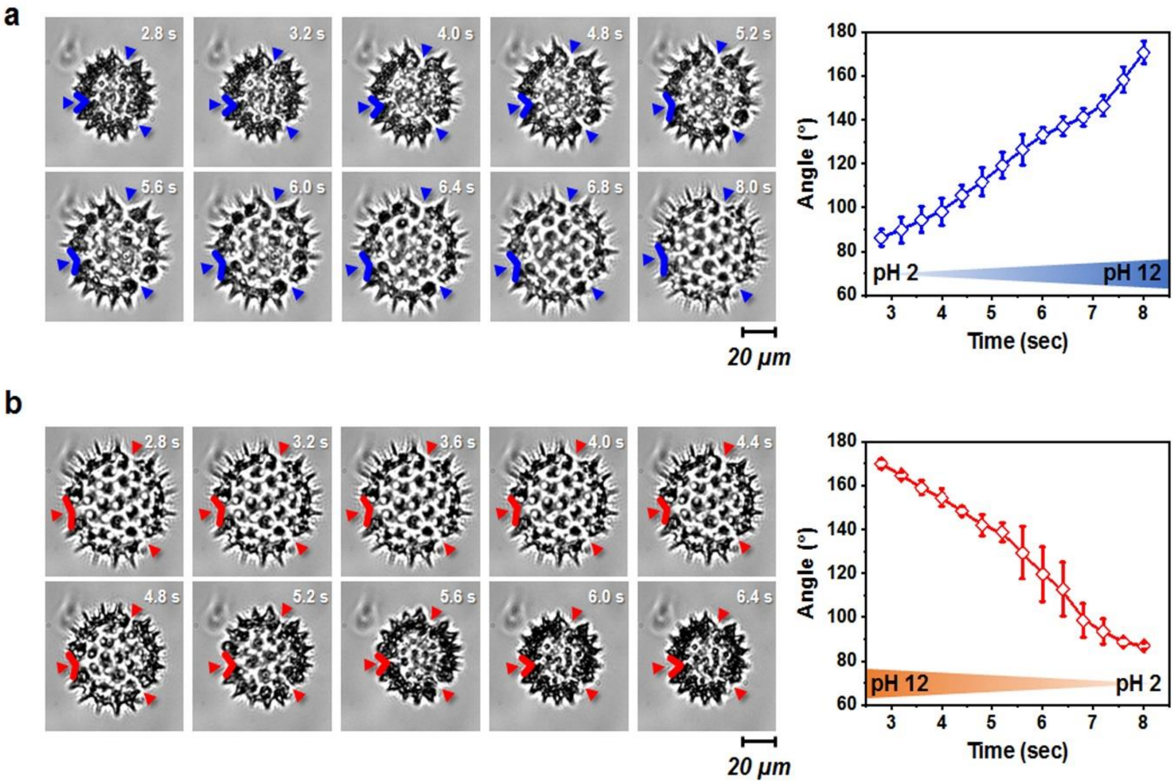

Supplementary Figure 8 | Time-lapse micrographs of 6 h KOH-treated sunflower pollen grains, responding to the gradual change of pH. a, When the solution pH was increased from 2 to 12, the apertures become larger due to swelling and exhibited a larger angle of opening. **b,** When the solution pH was decreased from 12 to 2, the apertures become smaller due to de-swelling and exhibited a smaller angle of opening. The average and standard deviations were obtained for 6 samples and the imaging interval was 0.4-s. Data are presented as mean ± s.d. Source data are provided as a Source Data file.

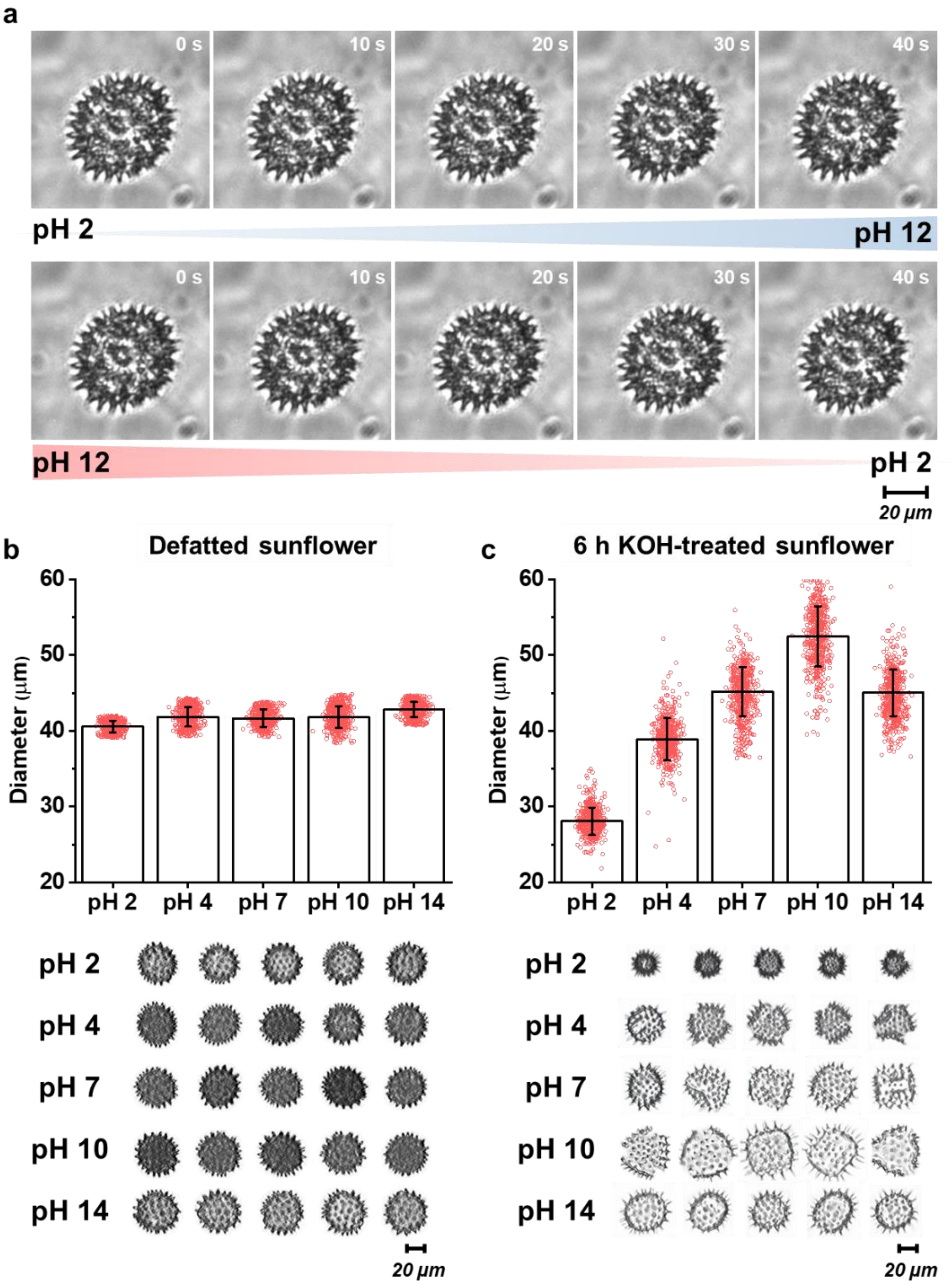

Supplementary Figure 9 | Characterization of the pH-dependent swelling behavior of defatted sunflower pollen without alkaline processing treatment. a, Time-lapse optical micrographs of defatted sunflower pollen particles tethered on a functionalized surface. The solution pH was changed from pH 2 to 12 (top series) and from pH 12 to 2 (bottom series). **b,** Top: Diameter of defatted sunflower pollen particles that did not undergo the alkaline processing protocol, as measured by DIPA (*n*=500 particles per condition). Bottom: Optical micrographs of representative, individual sunflower pollen grains in pH 2, 4, 7, 10, and 14 conditions at 10× magnification. **c,** Corresponding data for defatted sunflower pollen particles that underwent the alkaline processing protocol. Data are presented as mean ± s.d. Source data are provided as a Source Data file.

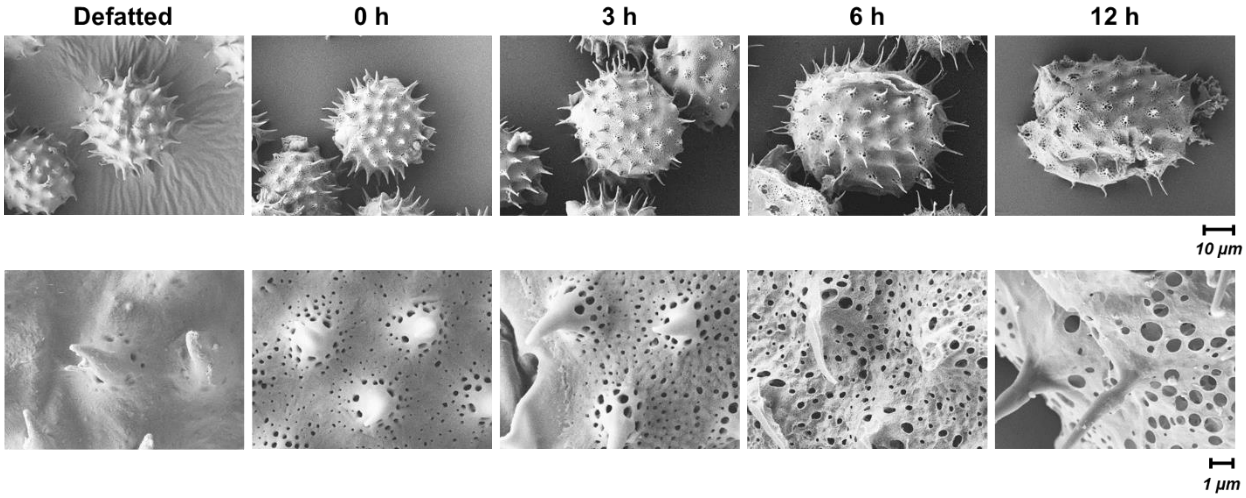

Supplementary Figure 10 | Scanning electron microscope (SEM) images of defatted sunflower pollen grains and pollen shells before and after 10% (wt/vol) KOH treatment. Top: SEM image of entire pollen microgels after defatting without or followed by 10% (wt/vol) KOH incubation (2nd KOH treatment) for 0–12 h. Longer KOH treatment resulted in greater opening of the apertures. Bottom: Surface morphology of exine layers at higher magnification. Exines of the defatted pollen grain exhibited a dense and smooth surface morphology with a few microscale pores around the spikes, while the exine surfaces of KOH-treated pollen were rough and porous with exposed sporopollenin skeleton. The increased porosity of the exine surface was attributed to the release of pollen cement ("pollenkitt") due to KOH treatment[11,12].

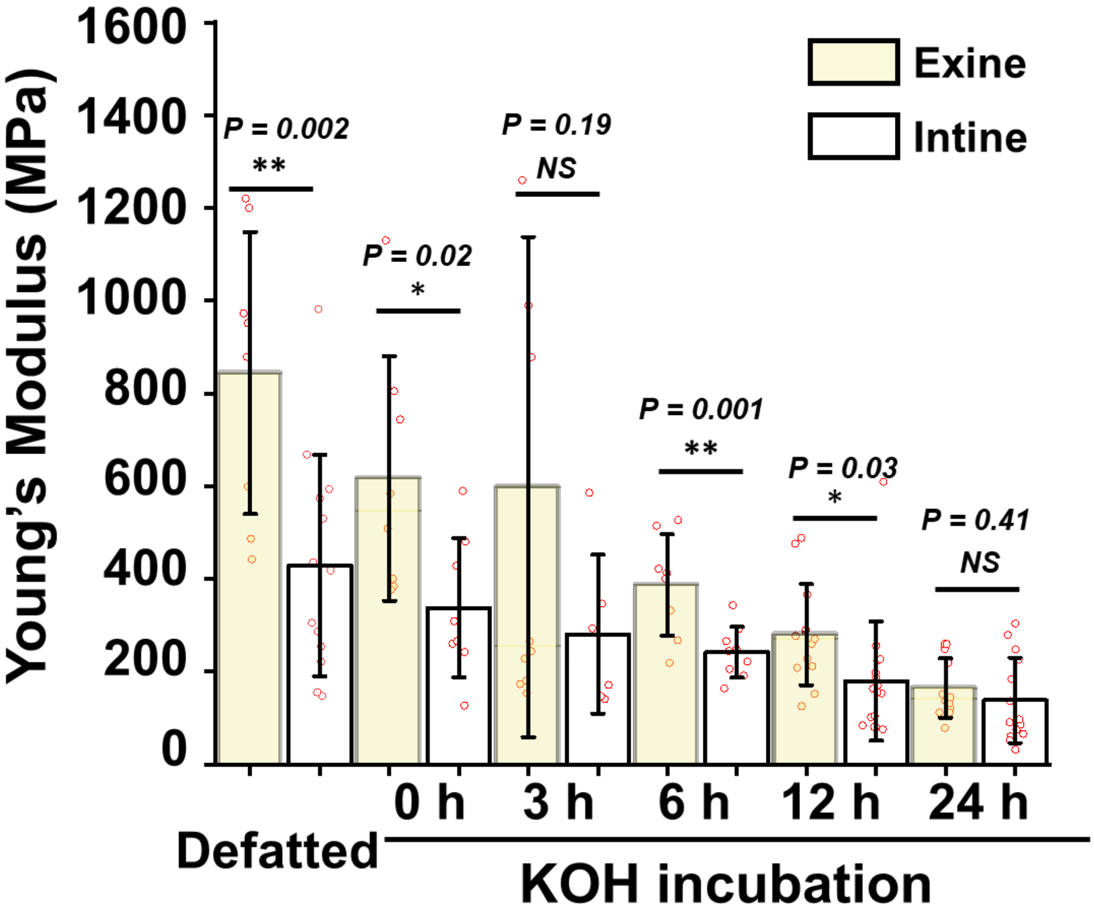

$\overline{M_{E/I}}$	2.7	2.2	2.8	1.7	1.6	1.2

Supplementary Figure 11 | AFM-characterized Young's modulus values of defatted sunflower pollen grains and pollen grains after treatment with 10% (wt/vol) KOH for various incubation times. Young's modulus values of exine and intine layers of various pollen microgels as a function of KOH incubation time. The fractured pollen microgels were fully hydrated in aqueous solution at pH 7 prior to measurement. Mean ± s.d. are reported with statistical analysis (n > 6, one-way analysis of variance (ANOVA) with Turkey's multiple comparisons test, *$P < 0.05$ and **$P < 0.01$). Source data are provided as a Source Data file.

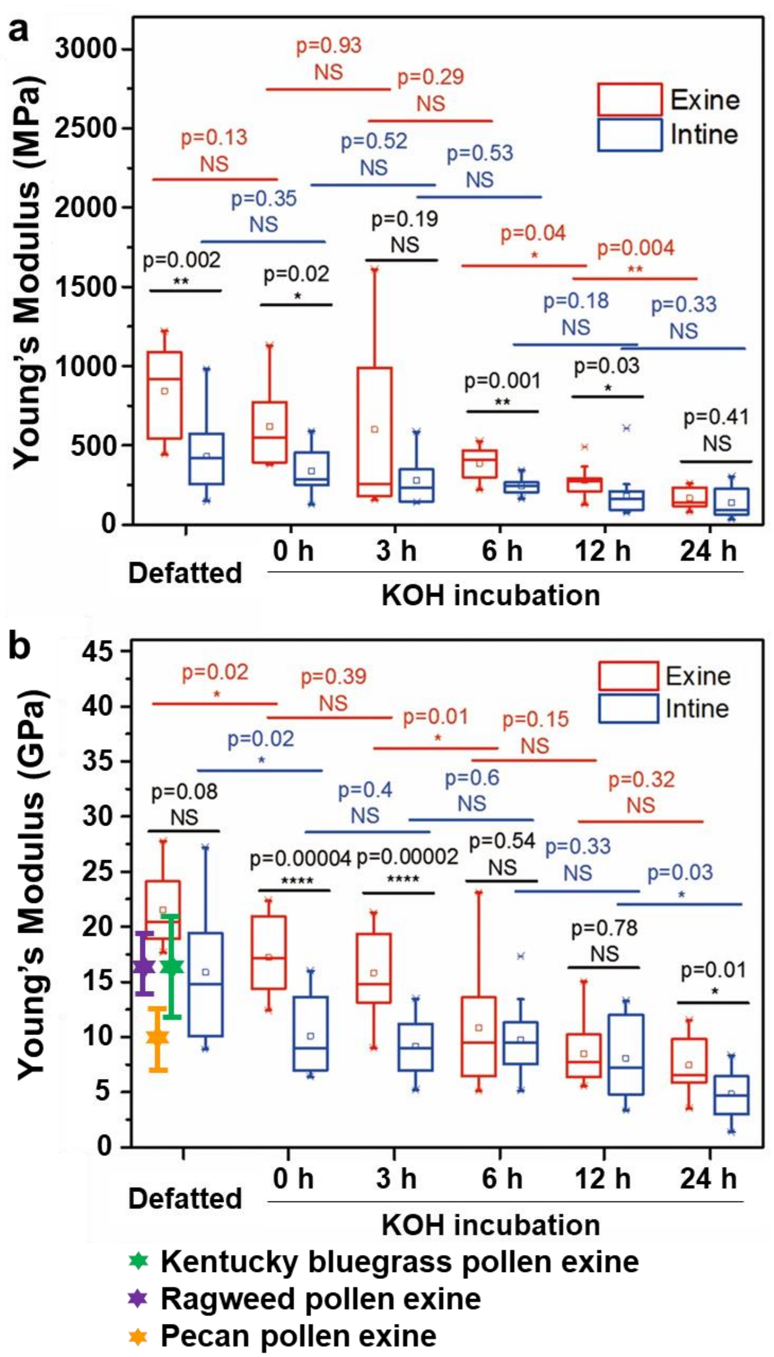

Supplementary Figure 12 | AFM-characterized Young's modulus values of defatted sunflower pollen grains and pollen grains after treatment with 10% (wt/vol) KOH for various incubation times. a, Young's modulus values of pollen grains in the wet condition. **b,** Young's modulus values of pollen grains in the dry condition. The Young's modulus values of defatted pollen grains in the dry condition are reported values from the literature[13,14], as indicated by star asterisks for Kentucky bluegrass, Ragweed, Pecan species. For both dry and wet conditions, with increasing KOH incubation time, the Young's modulus values of the exine and intine became significantly decreased, indicating mechanical degradation of the pollen shell. In the box plots, the boundary of the box closest to zero indicates the 25th percentile, a line within the box marks the

median, a square symbol within the box marks the mean, and the boundary of the box farthest from zero indicates the 75th percentile. Whiskers below and above the box indicate the 5th and 95th percentiles, respectively (n > 6, one-way analysis of variance (ANOVA) with Turkey's multiple comparisons test, *$P < 0.05$, **$P < 0.01$, ****$P < 0.0001$). Source data are provided as a Source Data file.

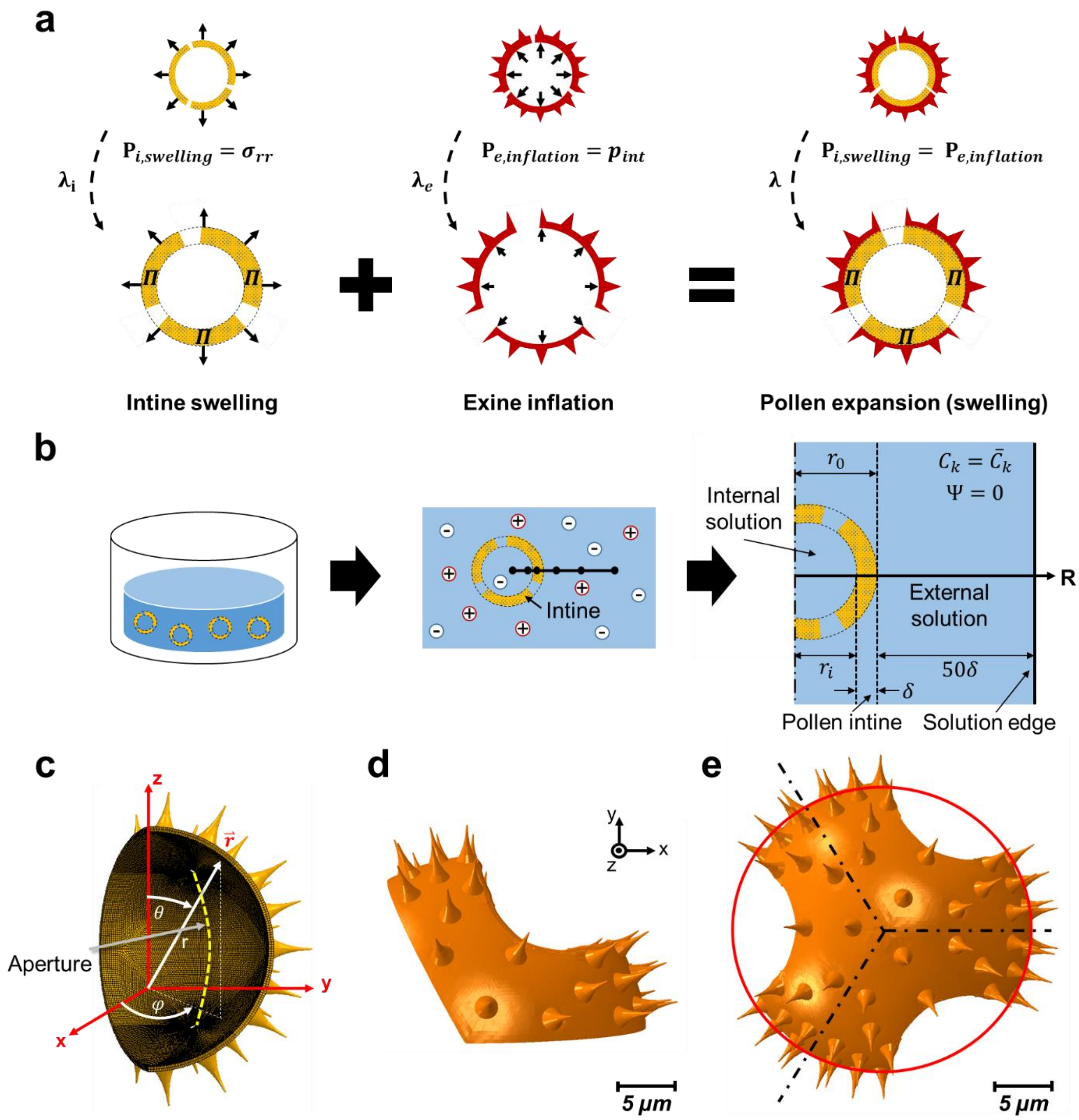

Supplementary Figure 13 | Multiphysics model of swelling of pollen microgel particles. **a,** Schematic illustration of pollen microgel swelling that is composed of the interplay of intine (hydrogel) swelling and exine (rubber) inflation. The swelling-induced mechanical pressure exerted on the intine layer ($P_{i,swelling}$) due to osmotic pressure effects (Π) decreases as intine swelling proceeds. The intine swelling pressure ($P_{i,swelling}$) is equal to the equilibrium pressure required to inflate the exine layer ($P_{e,inflation}$). The swelling ratio of the intine layer, λ_i, is equal to the inflation ratio of the exine layer, λ_e, due to the intine layer being tightly bound to the exine layer. The hydration and swelling of pollen microgels due to osmotic pressure-related effects were predicted based on the multiphysics model of the intine layer ($P_{i,swelling} = \sigma_{rr}$), and the pressure for exine inflation was applied to the internal surface of the exine layer using the boundary condition $\sigma_{rr} = p_{int}$, as proposed in Ref. 15. **b,** Schematics of the intine shell placed in an ionic

solution with the computational domain and boundary conditions for numerical simulations. **c**,. Geometrical configuration of pollen microgel particle shells used in the simulations, including exine layer and spikes. An appreciably finer mesh was placed on two tips of the aperture in order to avoid excessive mesh distortion during the aperture opening process. Note that the pollen microgel particle is modeled using one-third symmetry.*"* **d,** Deformed contour of a pollen grain simulated with one-third symmetry to effectively use computational resources. **e,** Measurement setup for the swollen diameter of a deformed pollen microgel particle structure. Note that the deformation is irregular and the red circle marks the maximum deformed diameter.

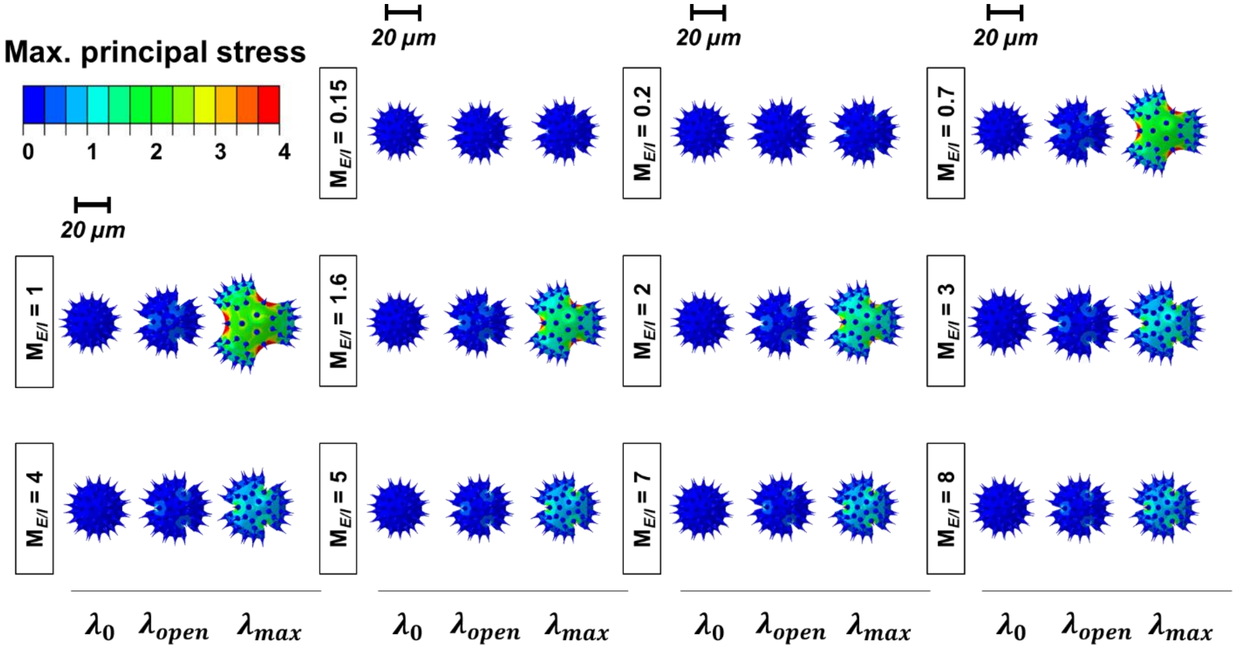

Supplementary Figure 14 | Maximum principal stress of the sunflower pollen microgels over various Young's modulus ratios ($M_{E/I}$ = 0.15, 0.2, 0.7, 1, 1.6, 2, 3, 4, 5, 7 and 8) at three critical swelling ratios (λ_0, λ_{open} and λ_{max}). $M_{E/I} < 1$ corresponds to the experimental results of pollen microgel particles exposed to various ion species with different concentrations. $M_{E/I} > 1$ corresponds to the experimental results of varied pH values. Typically, $M_{E/I} = 0.15$, $M_{E/I} = 0.2$, and $M_{E/I} = 0.7$ represent the 100 mM $CaCl_2$ solution, 10 mM $CaCl_2$, and 5 mM KCl solution conditions, respectively; $M_{E/I} = 1.6$ shows the 6 h KOH-treated pollen at pH 10 condition (as presented in Fig. 3a).

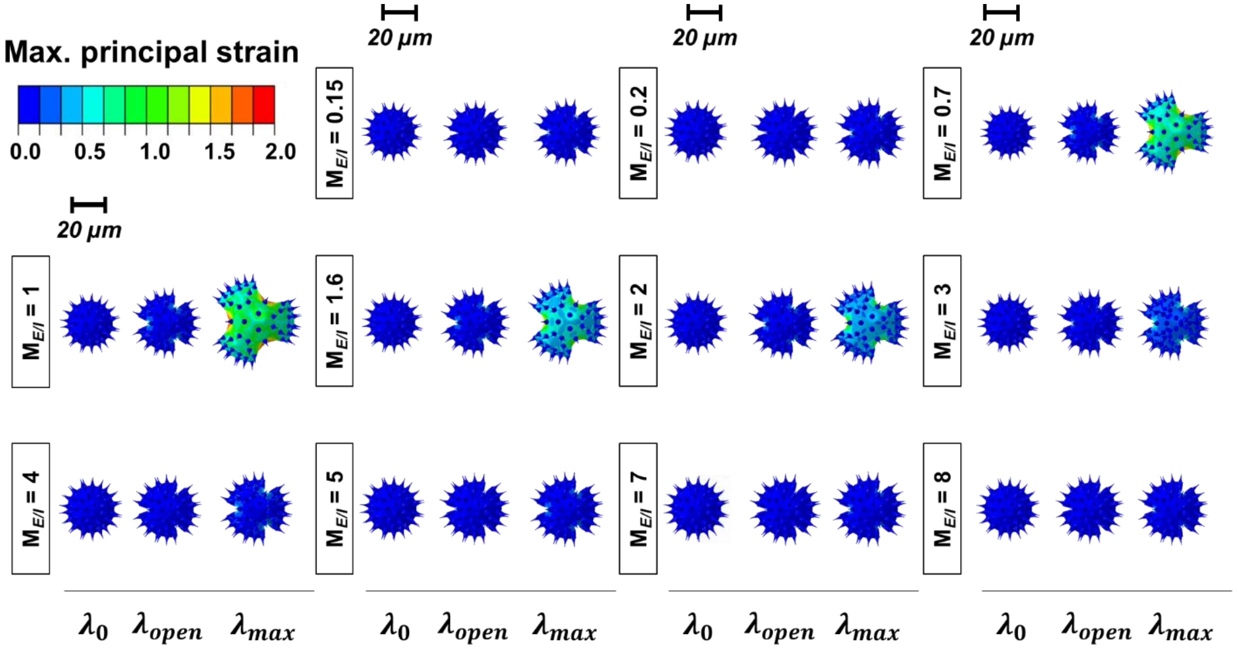

Supplementary Figure 15 | Maximum principal strain of the sunflower pollen microgel particles over various Young's modulus ratios ($M_{E/I}$ = 0.15, 0.2, 0.7, 1, 1.6, 2, 3, 4, 5, 7 and 8) at three critical swelling ratios (λ_0, λ_{open} and λ_{max}). $M_{E/I}$< 1 corresponds to the experimental results of pollen microgel particles exposed to various ion species with different concentrations. $M_{E/I} > 1$ corresponds to the experimental results of varied pH values. Typically, $M_{E/I} = 0.15$, $M_{E/I} = 0.2$, and $M_{E/I} = 0.7$ represent the 100 mM $CaCl_2$ solution, 10 mM $CaCl_2$, and 5 mM KCl solution conditions, respectively; $M_{E/I} = 1.6$ shows the 6 h KOH-treated pollen at pH 10 condition (as presented in Fig. 3a).

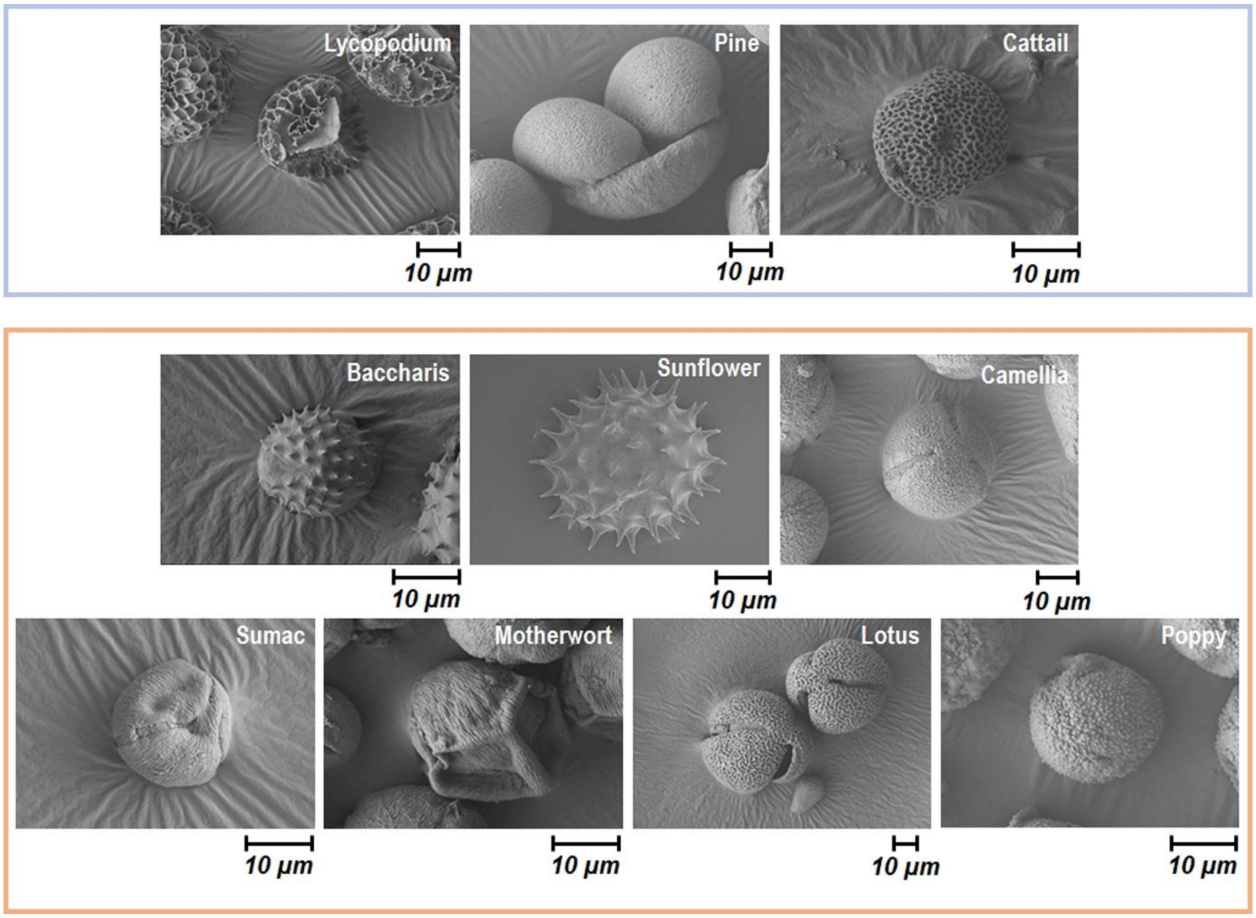

Supplementary Figure 16 | Characterization of various pollen grains by scanning electron microscope (SEM). Representative SEM images show the morphological properties of different defatted pollen species examined in this study.

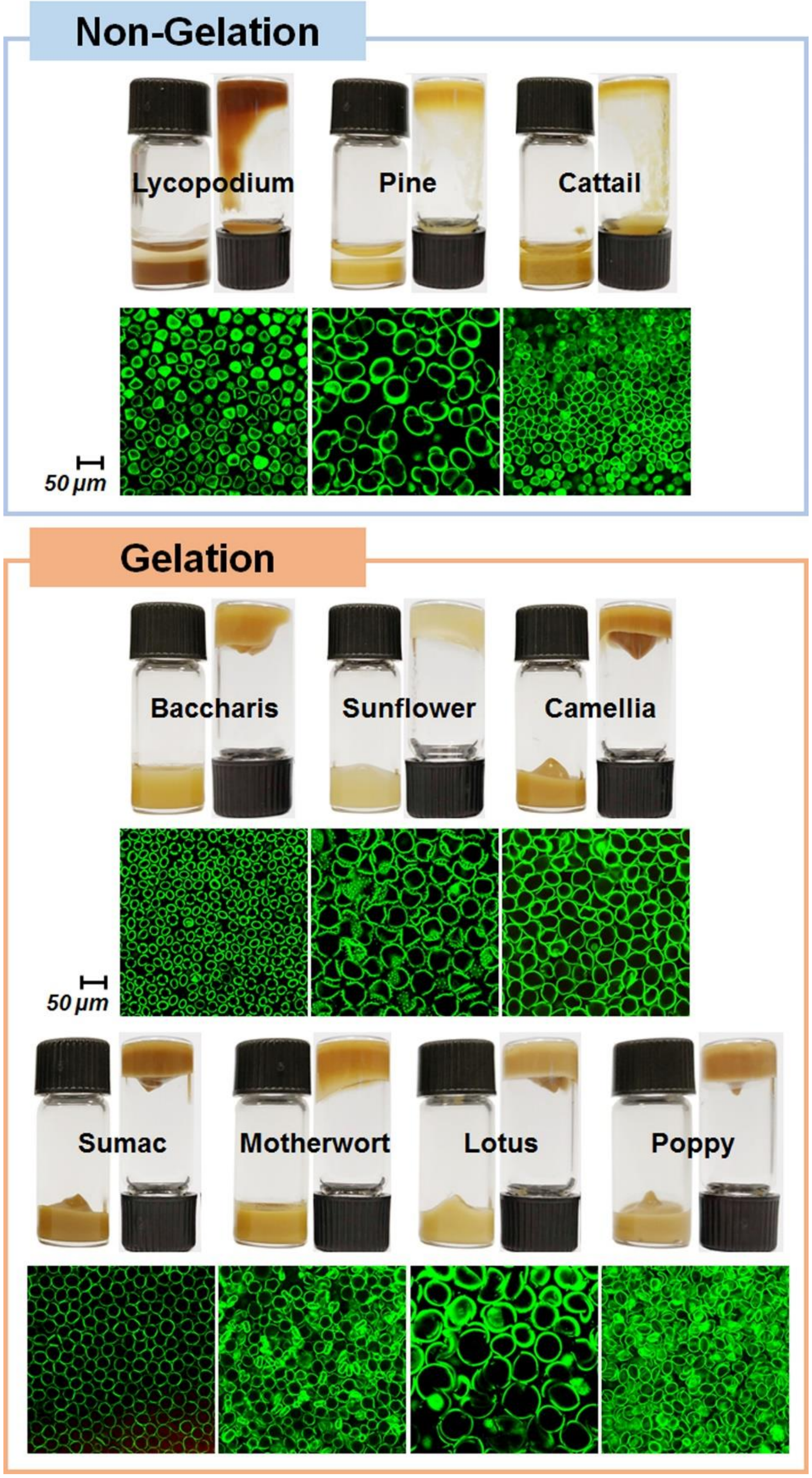

Supplementary Figure 17 | Cross-sectional confocal microscope images of 6 h KOH-treated pollen grains. Top: Lycopodium, pine, and cattail pollen particles do not show gelling properties. Bottom: Pollen from eudicot plants undergo gelation following 6 h incubation with 10% (wt/vol) KOH treatment.

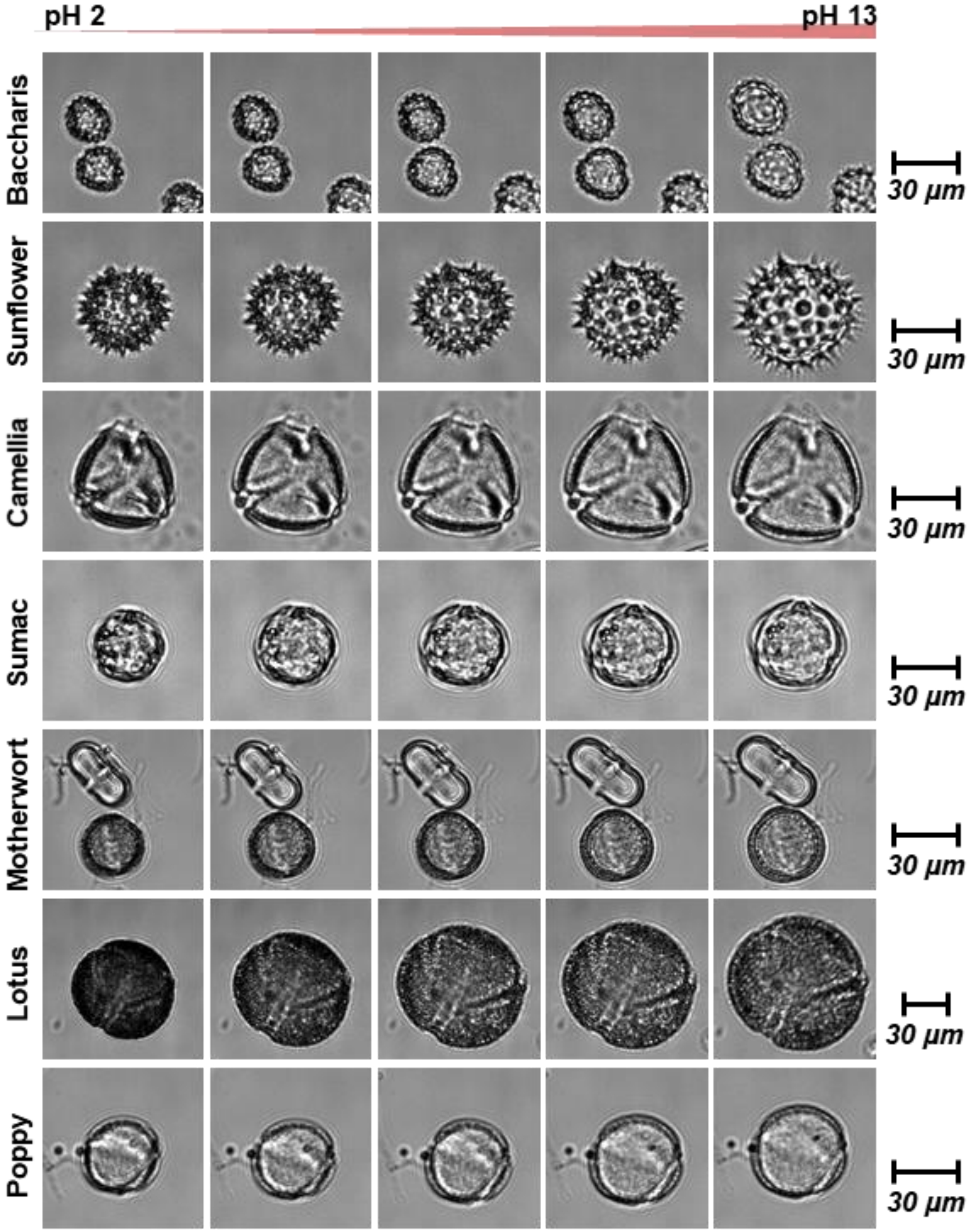

Supplementary Figure 18 | Time-lapse optical micrographs of various pollen species in response to changes in solution pH. Pollen species from the eudicot clade are responsive to pH change in solution over a short time span. Each image corresponds to a 0.4-s time interval in increasing order.

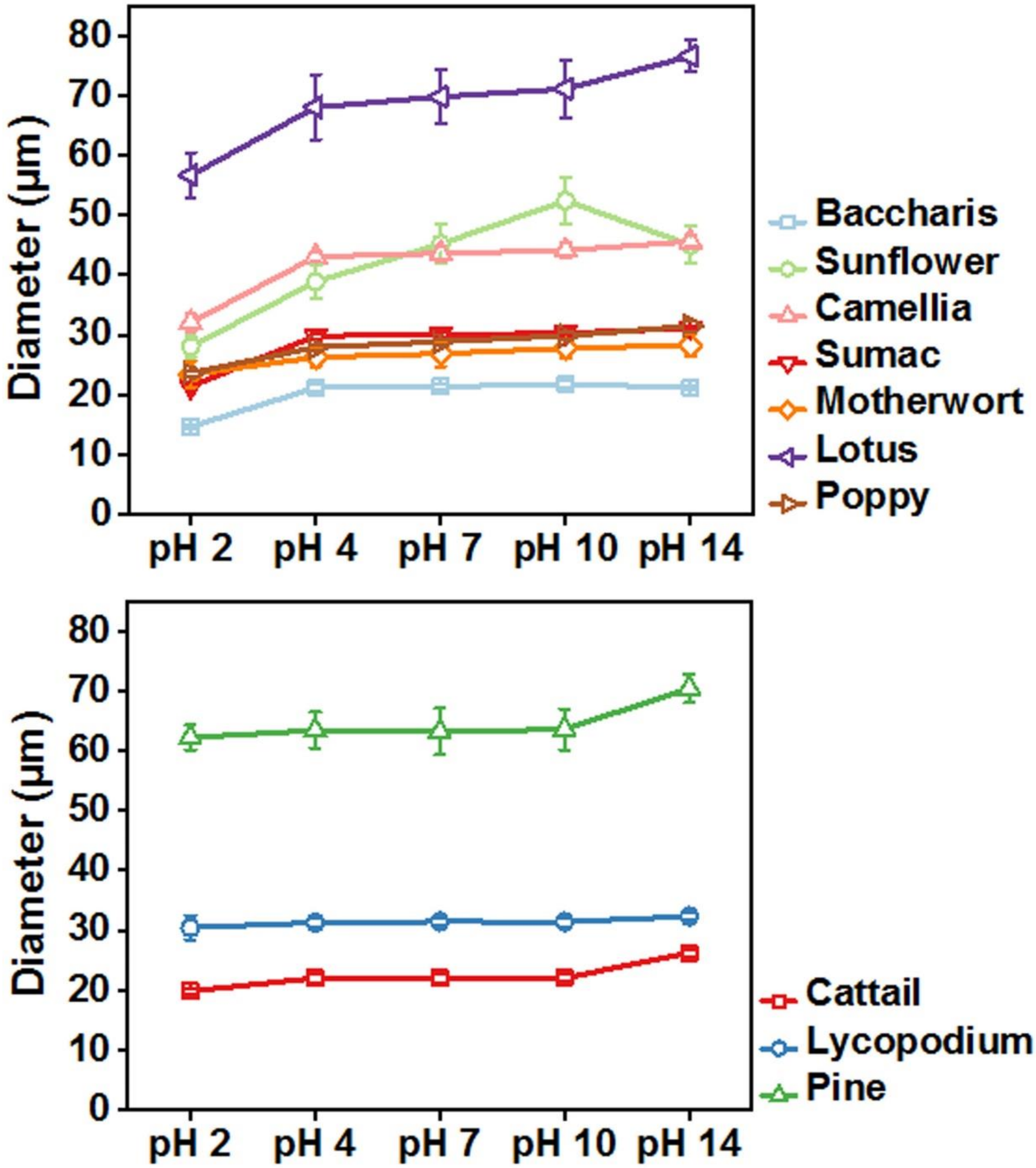

Supplementary Figure 19 | pH-responsive behavior of pollen grains from eudicots and other clades as observed by DIPA measurements. Top: Eudicot pollen species exhibit distinct pH-responsive behavior. Bottom: Pollen and spore species from the tested non-eudicot plants maintained similar size and shape in different pH environments. Data are reported as mean ± s.d. from n = 500 particles per condition. Source data are provided as a Source Data file.

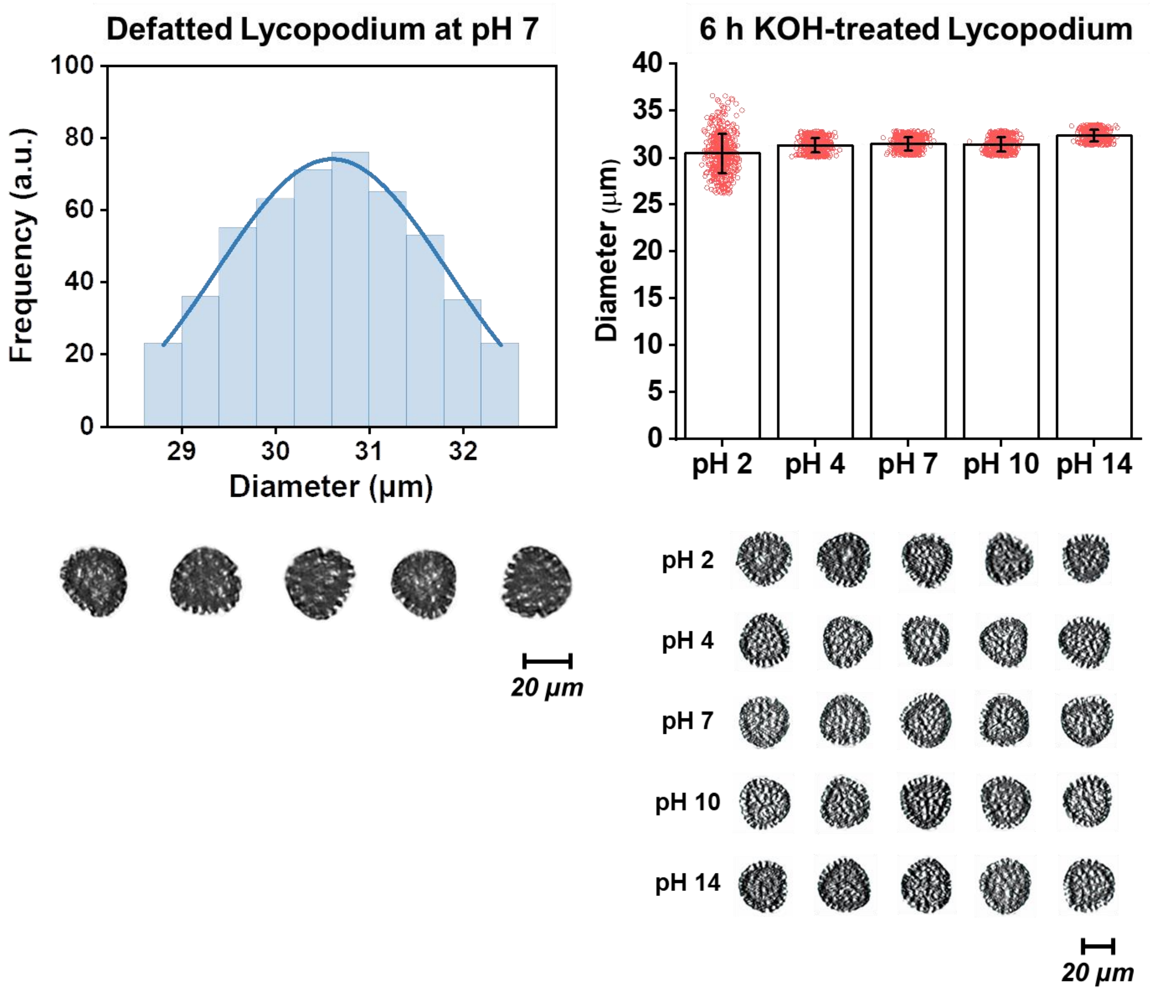

Supplementary Figure 20 | Characterization of defatted and 6 h KOH-treated lycopodium spores by DIPA measurements. Left: Size distribution of defatted spore particles before any KOH treatment. The size distribution was fitted to a Gaussian curve. Right: Microscopic characterization of 6 h KOH-treated defatted lycopodium spores in different pH conditions. Representative optical micrographs of spores were obtained at 10× magnification, and data are reported as mean ± s.d. from $n = 500$ particles per condition. Source data are provided as a Source Data file.

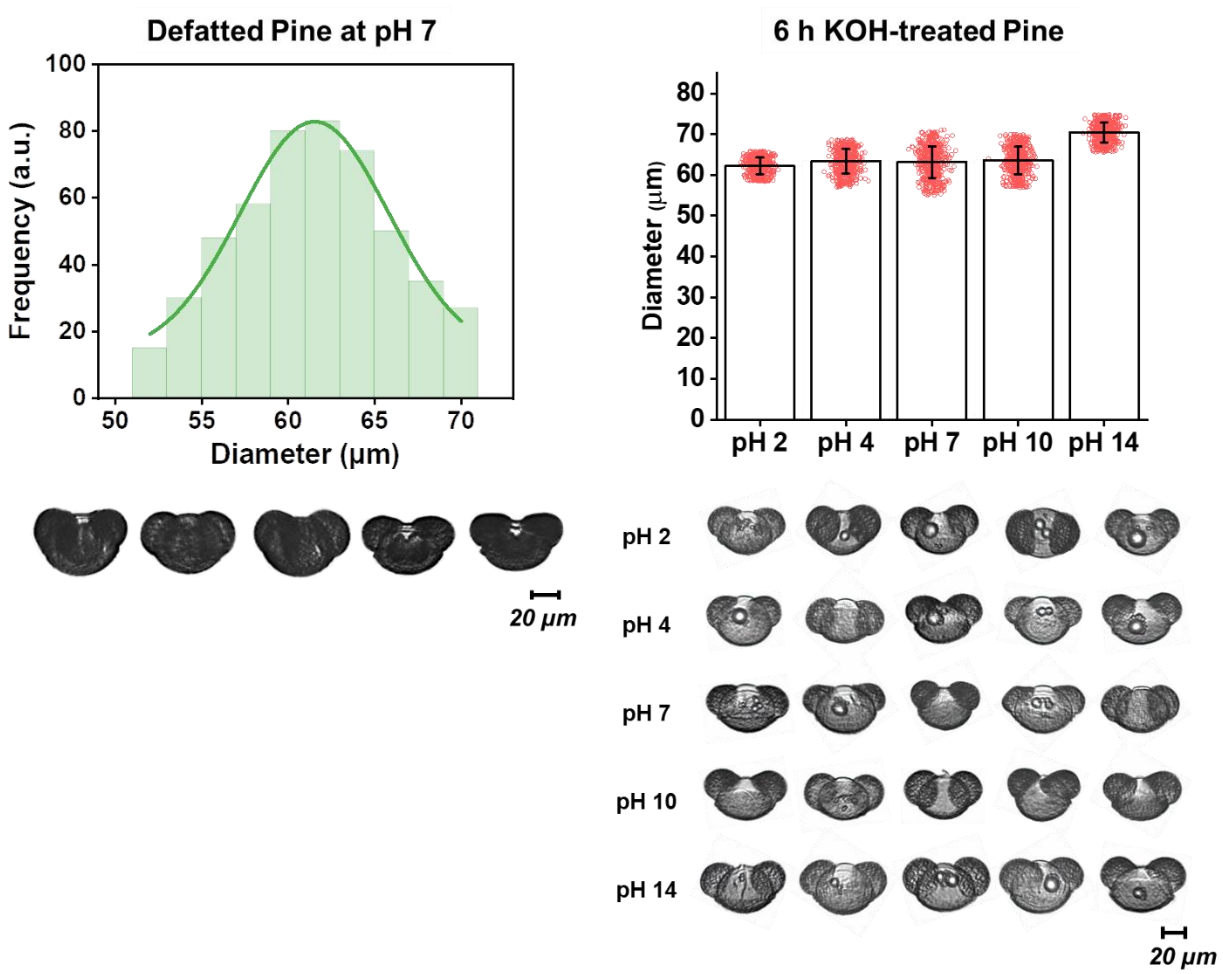

Supplementary Figure 21 | Characterization of defatted and 6 h KOH-treated pine pollen grains by DIPA measurements. Left: Size distribution of defatted pollen particles before any KOH treatment. The size distribution was fitted to a Gaussian curve. Right: Microscopic characterization of 6 h KOH-treated defatted pine pollen grains in different pH conditions. Representative optical micrographs of pollen grains were obtained at 10× magnification, and data are reported as mean ± s.d. from n = 500 particles per condition. Source data are provided as a Source Data file.

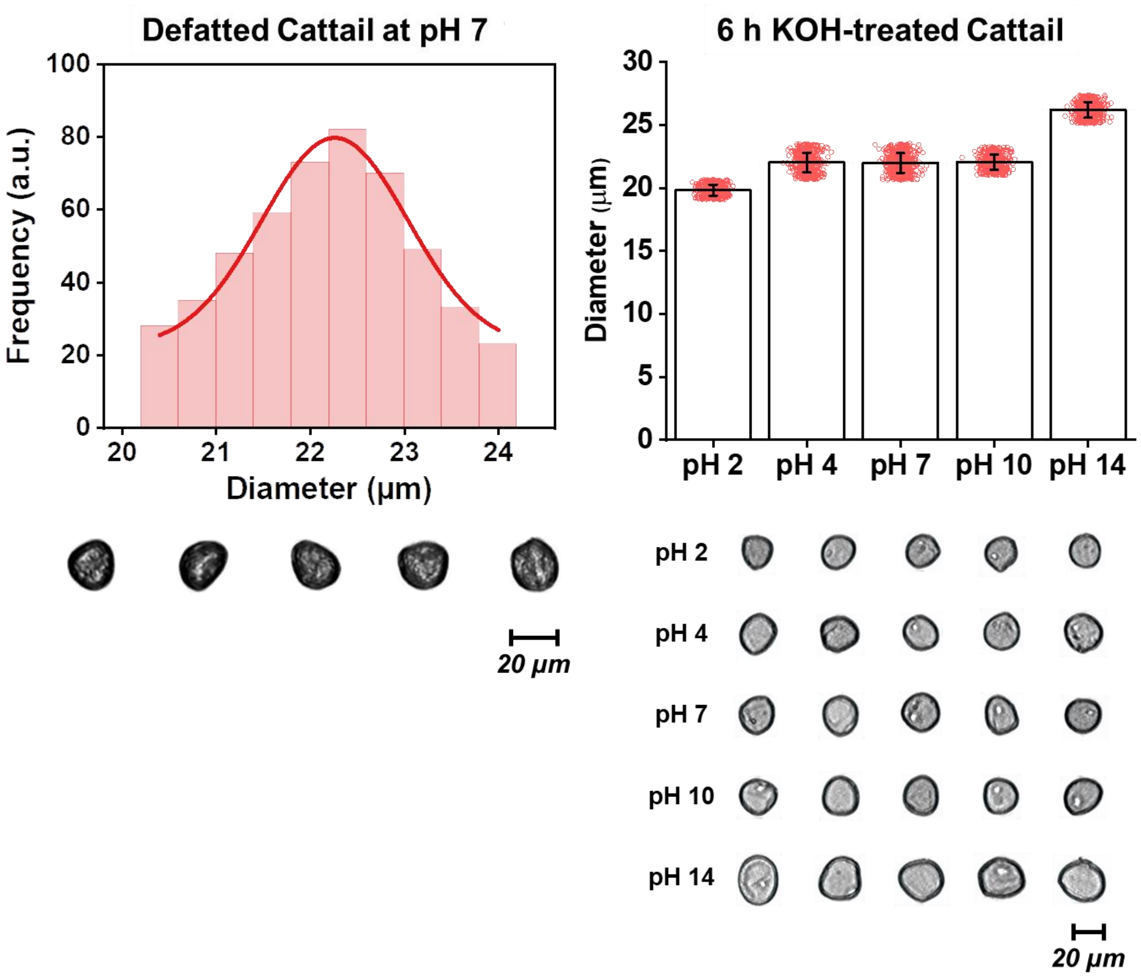

Supplementary Figure 22 | Characterization of defatted and 6 h KOH-treated cattail pollen grains by DIPA measurements. Left: Size distribution of defatted pollen particles before any KOH treatment. The size distribution was fitted to a Gaussian curve. Right: Microscopic characterization of 6 h KOH-treated defatted cattail pollen grains in different pH conditions. Representative optical micrographs of pollen grains were obtained at 10× magnification, and data are reported as mean ± s.d. from *n* = 500 particles per condition. Source data are provided as a Source Data file.

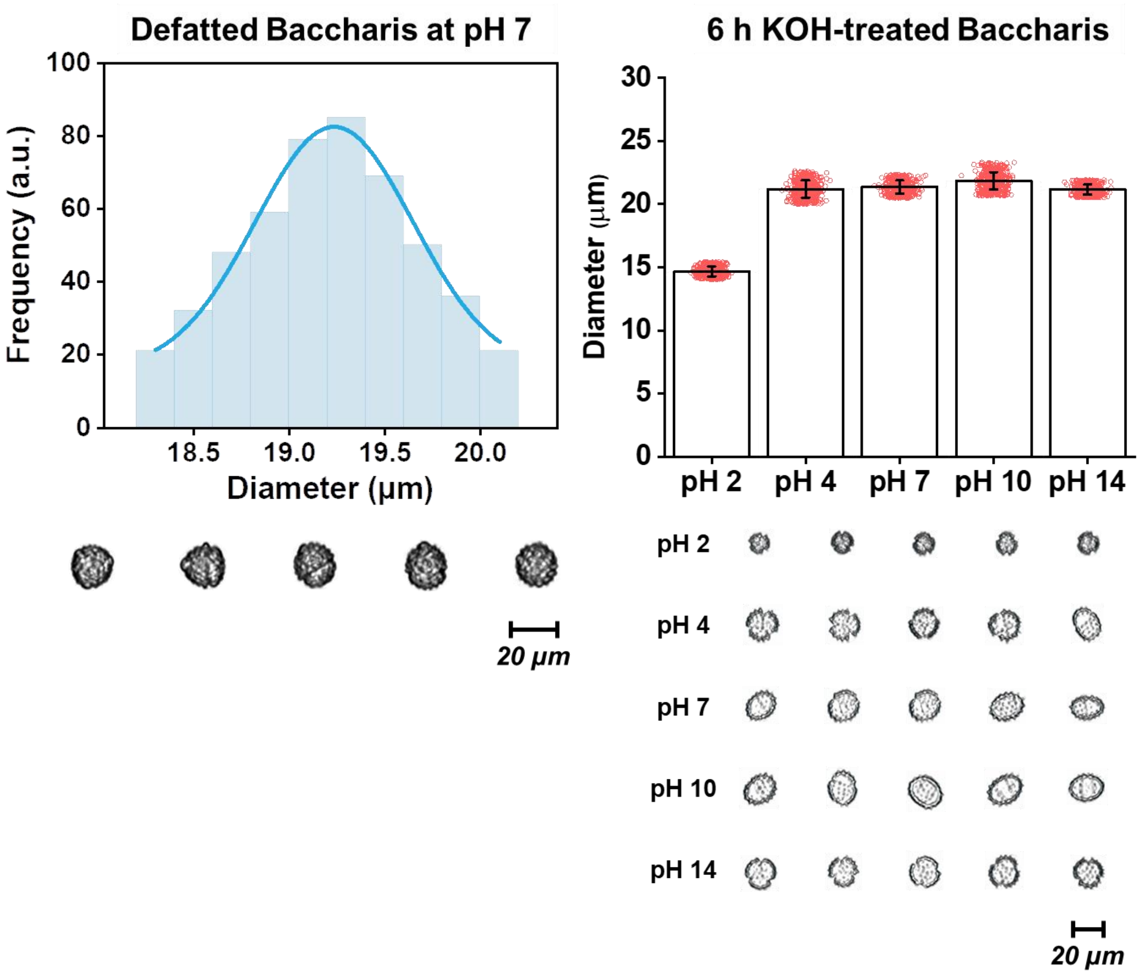

Supplementary Figure 23 | Characterization of defatted and 6 h KOH-treated baccharis pollen grains by DIPA measurements. Left: Size distribution of defatted pollen particles before any KOH treatment. The size distribution was fitted to a Gaussian curve. Right: Microscopic characterization of 6 h KOH-treated defatted baccharis pollen grains in different pH conditions. Representative optical micrographs of pollen grains were obtained at 10× magnification, and data are reported as mean ± s.d. from *n* = 500 particles per condition. Source data are provided as a Source Data file.

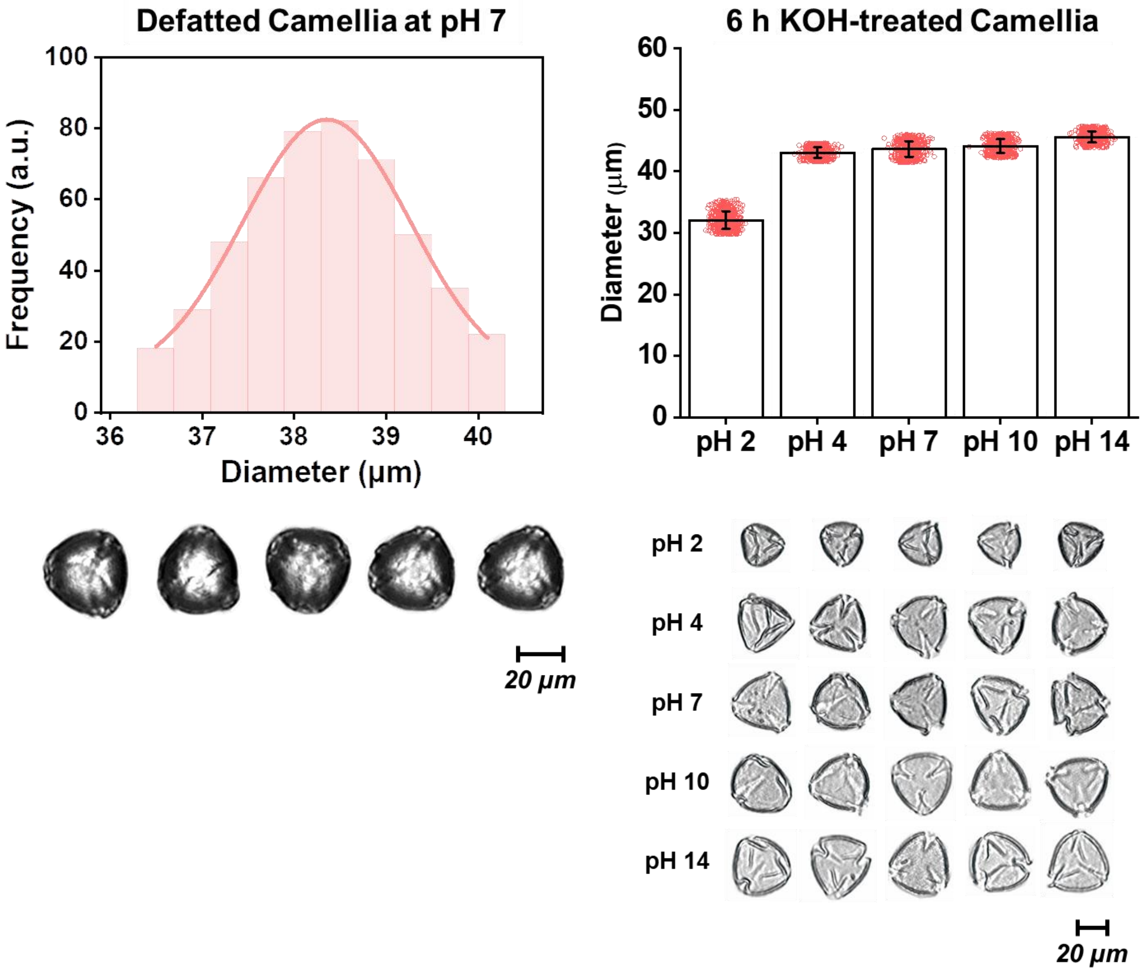

Supplementary Figure 24 | Characterization of defatted and 6 h KOH-treated camellia pollen grains by DIPA measurements. Left: Size distribution of defatted pollen particles before any KOH treatment. The size distribution was fitted to a Gaussian curve. Right: Microscopic characterization of 6 h KOH-treated defatted camellia pollen grains in different pH conditions. Representative optical micrographs of pollen grains were obtained at 10× magnification, and data are reported as mean ± s.d. from n = 500 particles per condition. Source data are provided as a Source Data file.

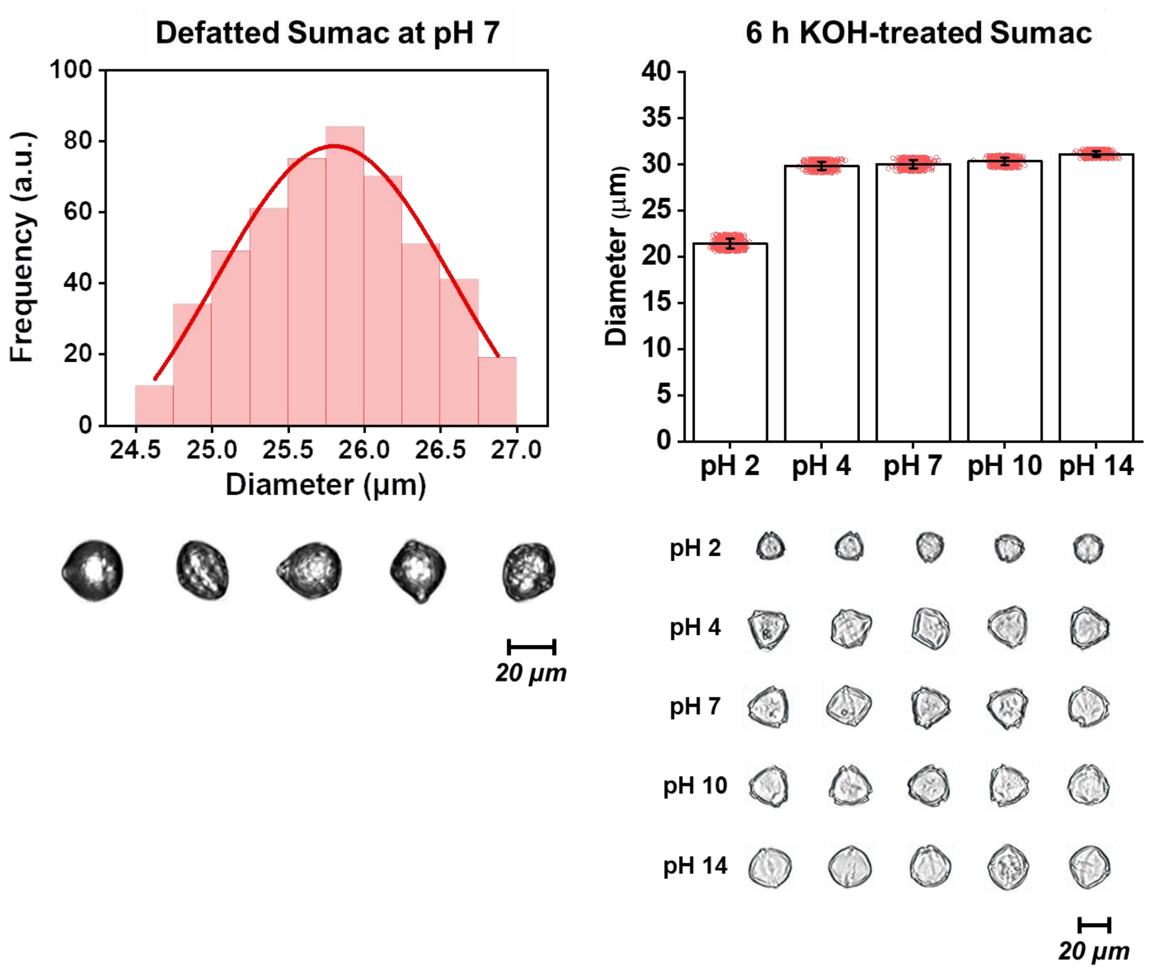

Supplementary Figure 25 | Characterization of defatted and 6 h KOH-treated sumac pollen grains by DIPA measurements. Left: Size distribution of defatted pollen particles before any KOH treatment. The size distribution was fitted to a Gaussian curve. Right: Microscopic characterization of 6 h KOH-treated defatted sumac pollen grains in different pH conditions. Representative optical micrographs of pollen grains were obtained at 10× magnification, and data are reported as mean ± s.d. from n = 500 particles per condition. Source data are provided as a Source Data file.

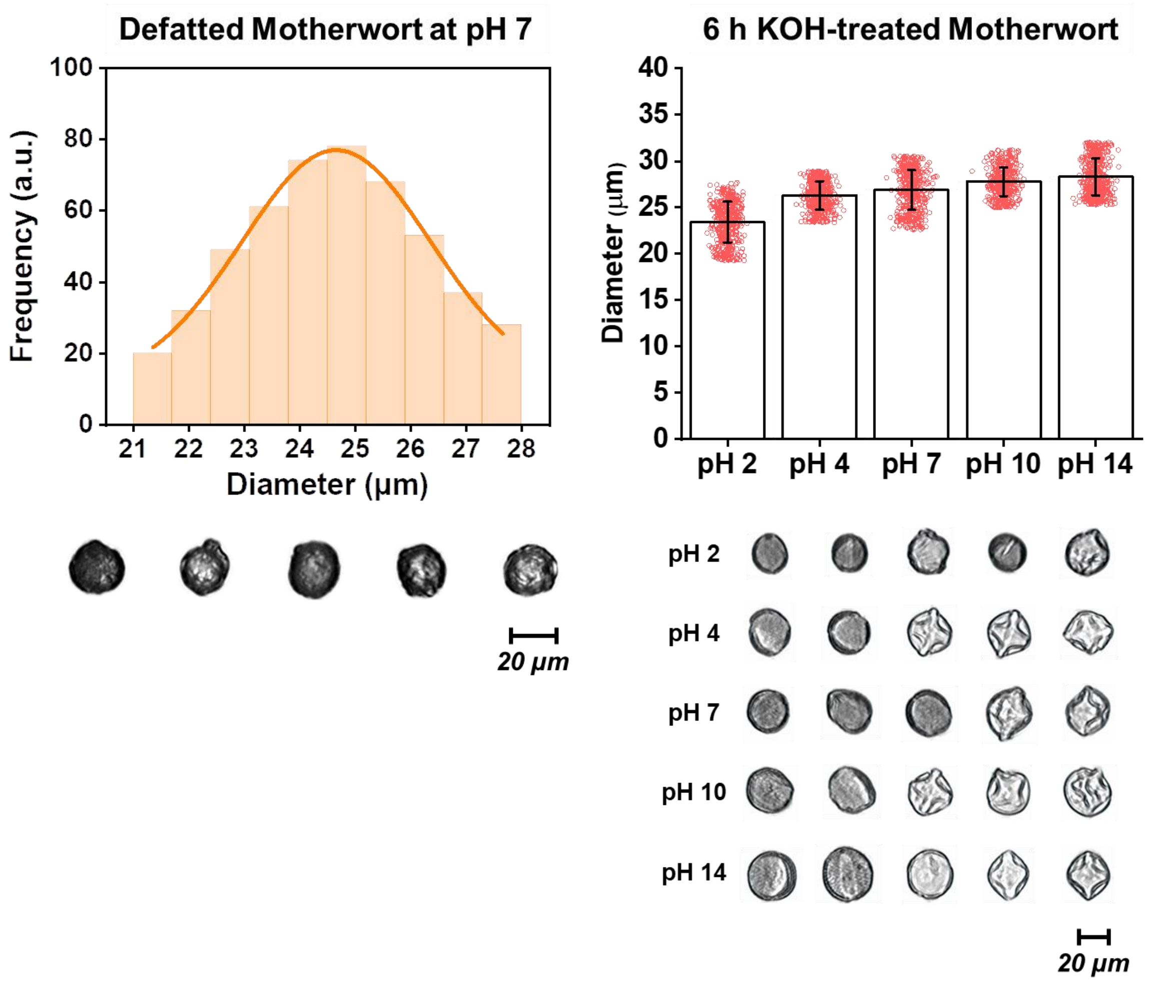

Supplementary Figure 26 | Characterization of defatted and 6 h KOH-treated motherwort pollen grains by DIPA measurements. Left: Size distribution of defatted pollen particles before any KOH treatment. The size distribution was fitted to a Gaussian curve. Right: Microscopic characterization of 6 h KOH-treated defatted motherwort pollen grains in different pH conditions. Representative optical micrographs of pollen grains were obtained at 10× magnification, and data are reported as mean ± s.d. from n = 500 particles per condition. Source data are provided as a Source Data file.

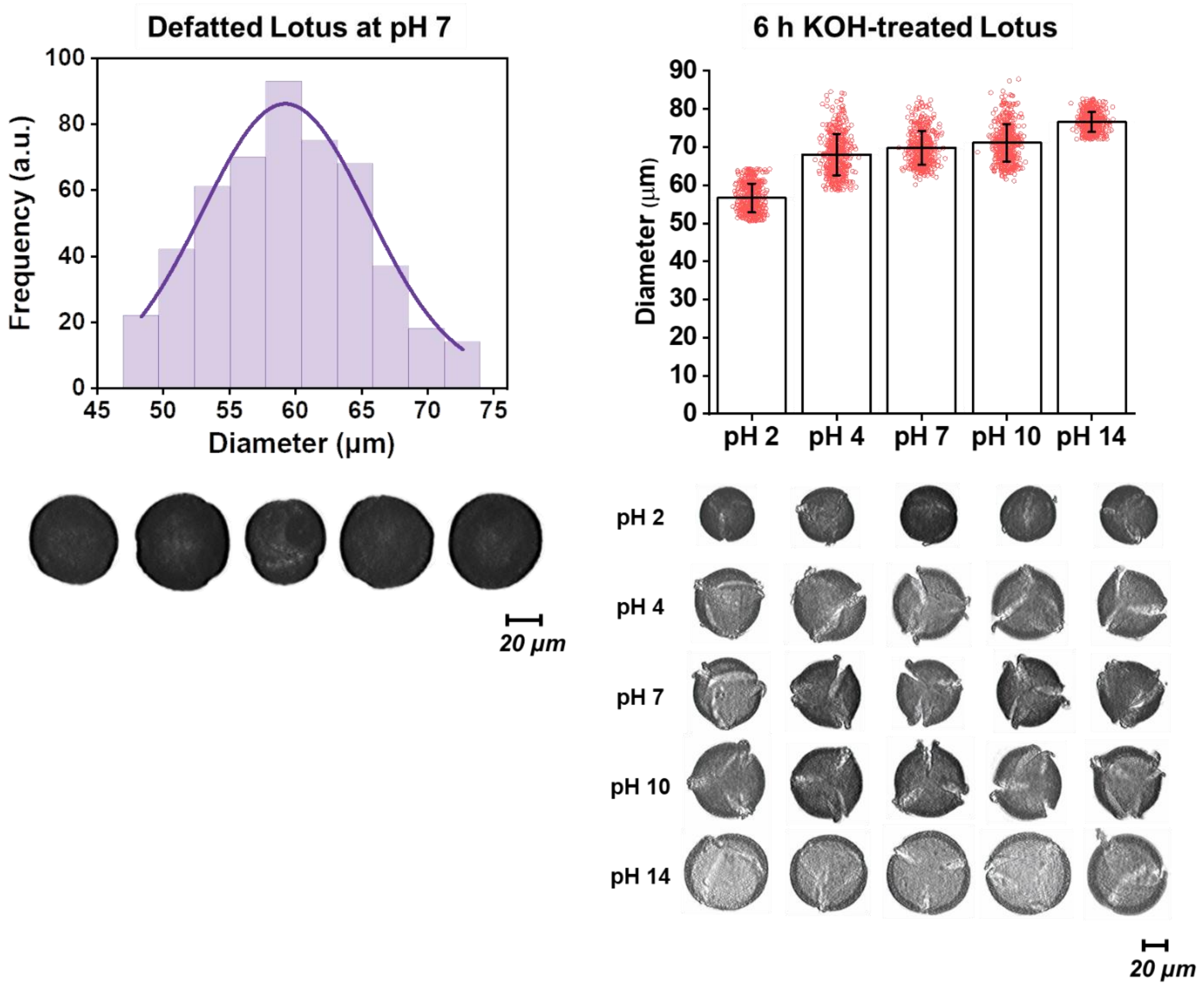

Supplementary Figure 27 | Characterization of defatted and 6 h KOH-treated lotus pollen grains by DIPA measurements. Left: Size distribution of defatted pollen particles before any KOH treatment. The size distribution was fitted to a Gaussian curve. Right: Microscopic characterization of 6 h KOH-treated defatted lotus pollen grains in different pH conditions. Representative optical micrographs of pollen grains were obtained at 10× magnification, and data are reported as mean ± s.d. from n = 500 particles per condition. Source data are provided as a Source Data file.

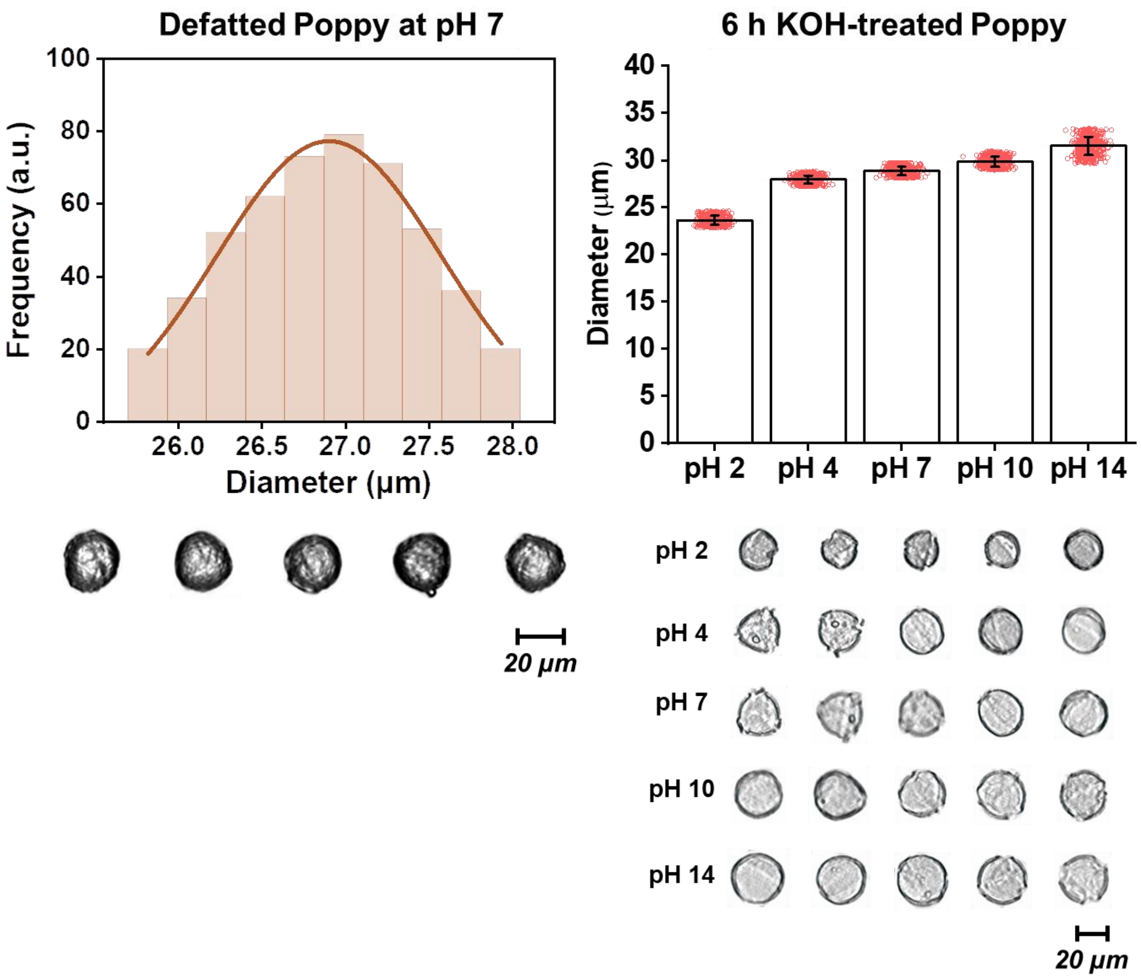

Supplementary Figure 28 | Characterization of defatted and 6 h KOH-treated poppy pollen grains by DIPA measurements. Left: Size distribution of defatted pollen particles before any KOH treatment. The size distribution was fitted to a Gaussian curve. Right: Microscopic characterization of 6 h KOH-treated defatted poppy pollen grains in different pH conditions. Representative optical micrographs of pollen grains were obtained at 10× magnification, and data are reported as mean ± s.d. from n = 500 particles per condition. Source data are provided as a Source Data file.

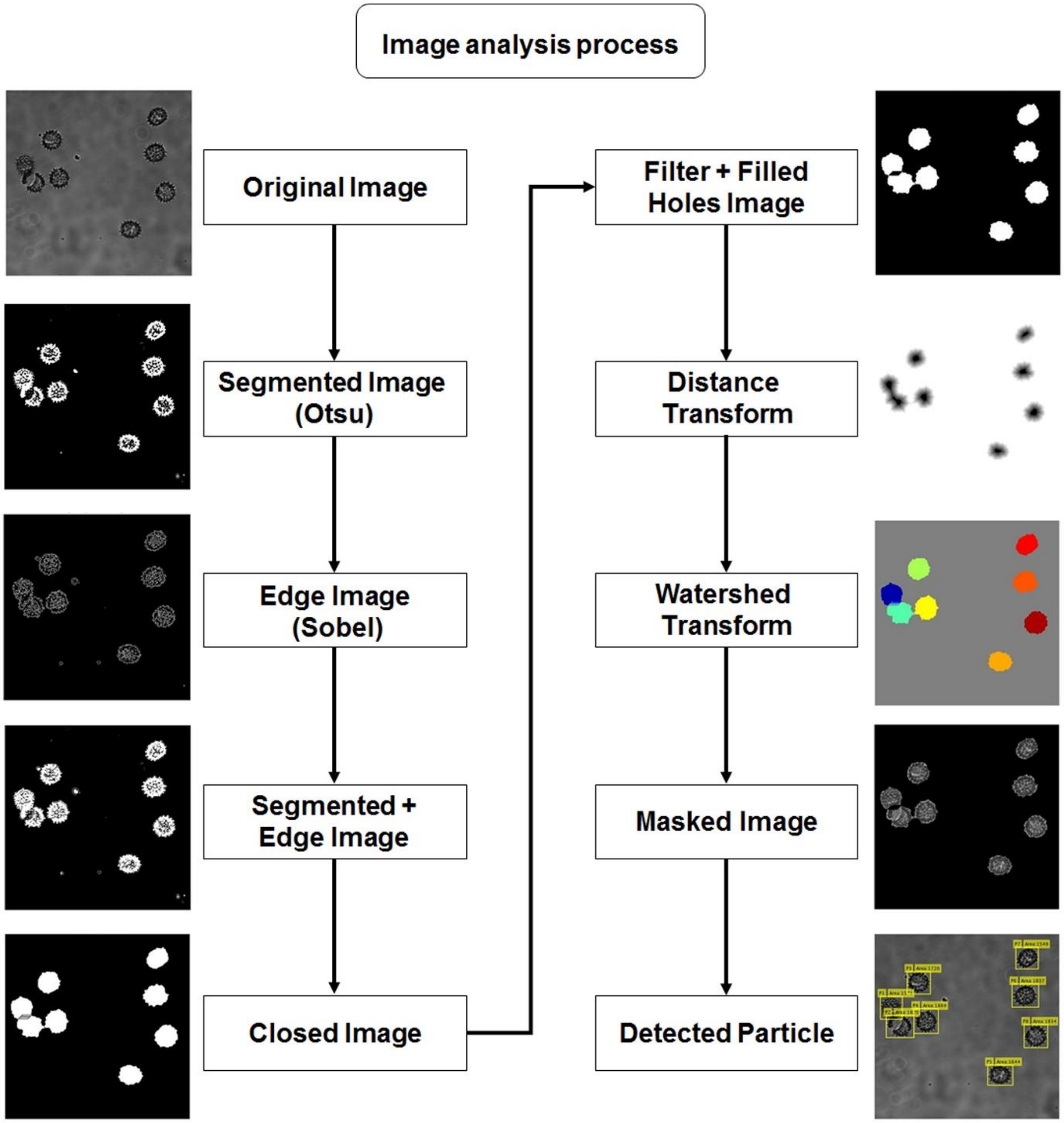

Supplementary Figure 29 | Image analysis procedure to measure the area of pollen grains. A series of processing steps were undertaken on original microscopy images using an in-house built MATLAB code utilizing a computer vision system toolbox.

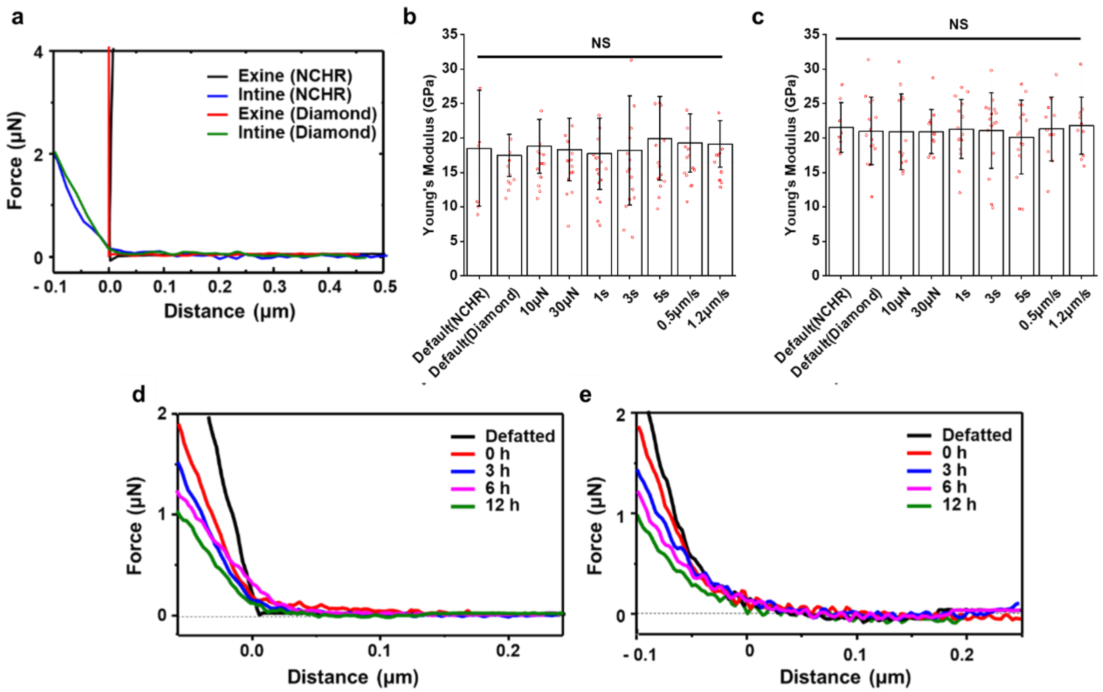

Supplementary Figure 30 | Effects of various AFM parameters on Young's modulus measurement of exine and intine layers of dry, defatted pollen grains. a, Representative force-displacement curves from AFM measurements using two types of AFM probes, an aluminum reflex-coated silicon cantilever PPP-NCHR (Nanosensors, Neuchâtel, Switzerland) with a typical spring constant of ~42 N m^{-1} and a tip end radius of 5 nm and a diamond cantilever TD26135 (Micro Star Technology, Texas, USA) with a spring constant of ~150 N m^{-1} and a tip end radius of ~5 nm. **b** and **c,** Young's moduli of exine (b) and intine (c) retrieved from force-displacement curves after AFM measurements using two AFM probes, NCHR and diamond probes at a maximum contact force of 20 μN with setpoint of 6 μN with zero contact time and an approach speed of 0.8 μN s^{-1}. AFM measurements were performed using the NCHR probe, varying the maximum contact force (10 to 30 μN), contact time (0 to 1, 3, and 5 s), and approach speed (0.5 to 1.2 μN s^{-1}). Data are reported as mean ± s.d. from $n > 6$ particles per condition (one-way analysis of variance (ANOVA) with Turkey's multiple comparisons test). **d,** Representative force-displacement curves of exine layer measurements in the wet condition. **e,** Representative force-displacement curves of intine layer measurements in the wet condition. Source data are provided as a Source Data file.

3. Supplementary Tables (1-2)

Supplementary Table 1 | Ratios of exine to intine Young's modulus values for the parametric study on the swelling behavior of pollen microgel particles. Based on the modulus data of the exine (E_e) and intine (E_i) layers that were obtained in the AFM measurements, we calculated the Young's modulus ratio ($M_{E/I}$) depending on the treatment conditions of the microgel particles during material processing. We performed a parametric study by varying $M_{E/I}$ from 0.15 to 8, as indicated in the table below. Verification and validation of the simulation models were implemented with respect to the swelling ratio based on a reference model for $M_{E/I}$ of 1.6 (grey highlighted column in the table). When the applied pressure was 8.3 MPa, a simulated swelling ratio of 1.80 (associated with a swollen diameter of 50.4 μm) was observed, showing good agreement with swelling data for 6 h KOH-treated pollen at pH 10 condition. Other conditions, such as $M_{E/I}$ of ≥ 2.0 or ≤ 0.5 represented pollen particles treated for shorter KOH incubation times (0 to 3 h) or exposed to different concentrations of divalent cations, respectively.

Modulus of the exine ($E_e \backslash MPa$)	400	400	400	250	400	500	750	1000	1250	1750	2000
Modulus of the intine ($E_i \backslash MPa$)	2609	2003	567	250	250	250	250	250	250	250	250
Modulus ratio ($M_{E/I}$= E_e/E_i)	0.15	0.2	0.7	1	1.6	2	3	4	5	7	8
Internal pressure (MPa)	1.6	2	8	8.3	8.3	8.3	8.3	8.3	8.3	8.3	8.3

Supplementary Table 2 | Input parameters for numerical simulations of the intine layer shell.

**Parameter**	**Value**
Relative dielectric permittivity ε_r	80
Young's modulus E ($\mathrm{pH}=7$)	250 MPa
Poisson's ratio υ	0.475
Flory-Huggins interaction parameter χ_H	0.1
Radius of the pollen intine at dry state r_0	13.42 μm
Thickness of the pollen intine at dry state δ	0.54 μm
Dissociation constant K	0.1 mM
External ionic concentration $\overline{C}_{H^+}$	$10^{(3-\mathrm{pH})}$ mM
External ionic concentration $\overline{C}_{OH^-}$	$10^{(\mathrm{pH}-11)}$ mM
External ionic concentration $\overline{C}_k$	Depending on the solution

4. Supplementary References

1 Nikonenko, V., Lebedev, K., Manzanares, J. & Pourcelly, G. Modelling the transport of carbonic acid anions through anion-exchange membranes. *Electrochimica Acta* **48**, 3639-3650, doi:10.1016/S0013-4686(03)00485-7 (2003).

2 Li, H., Luo, R., Birgersson, E. & Lam, K. Y. A chemo-electro-mechanical model for simulation of responsive deformation of glucose-sensitive hydrogels with the effect of enzyme catalysis. *Journal of the Mechanics and Physics of Solids* **57**, 369-382, doi:10.1016/j.jmps.2008.10.007 (2009).

3 Lai, F. & Li, H. Transient modeling of the reversible response of the hydrogel to the change in the ionic strength of solutions. *Mech. Mater.* **43**, 287-298 (2011).

4 Hong, W., Zhao, X., Zhou, J. & Suo, Z. A theory of coupled diffusion and large deformation in polymeric gels. *J. Mech. Phys. Solids* **56**, 1779-1793 (2008).

5 Hong, W., Zhao, X. & Suo, Z. Large deformation and electrochemistry of polyelectrolyte gels. *J. Mech. Phys. Solids* **58**, 558-577 (2010).

6 Pilkey, W. D. *Formulas for stress, strain, and structural matrics*. (Wiley, New York, 1994).

7 Bergström, J. *Mechanics of solid polymers: Theory and computational modeling*. (William Andrew, 2015).

8 ABAQUS. *ABAQUS 2017 User documentation*. (Dassault Systemes Simulia Corp., 2017).

9 Faye, A., Rodríguez-Martínez, J. & Volokh, K. Spherical void expansion in rubber-like materials: The stabilizing effects of viscosity and inertia. *Int. J. Nonlin. Mech.* **92**, 118-126 (2017).

10 Willats, W. G. *et al.* Analysis of pectic epitopes recognised by hybridoma and phage display monoclonal antibodies using defined oligosaccharides, polysaccharides, and enzymatic degradation. *Carbohydr. Res.* **327**, 309-320 (2000).

11 Heslop-Harrison, J. Pollen germination and pollen-tube growth. *Int. Rev. Cytol.* **107**, 1-78 (1980).

12 Pacinia, E. & Hesseb, M. Pollenkitt – Its composition, forms and functions. *Flora* **200**, 399–415 (2005).

13 Huang, C., Wang, Z., Quinn, D., Suresh, S. & Hsia, K. J. Differential growth and shape formation in plant organs. *Proc. Natl. Acad. Sci. U. S. A.* **115**, 12359-12364 (2018).

14 Qu, Z. & Meredith, J. C. The atypically high modulus of pollen exine. *J. R. Soc. Interface* **15** (2018).

15 Liu, X. *et al.* Ingestible hydrogel device. *Nat. Commun.* **10** (2019).
