# Supplementary material for: Transformation of hard pollen into soft matter
Source: Nat Commun. 2020 Mar 19;11:1449. doi: 10.1038/s41467-020-15294-w (PMC7081183; doi:10.1038/s41467-020-15294-w)
Supplement: Supplementary file 3 — Description of Additional Supplementary Files [file 41467_2020_15294_MOESM3_ESM.docx]

**Description of Additional Supplementary Files**

**Title: Supplementary Movie 1.** Microscopic observation of tethered single sunflower pollen grain in aqueous solution. Over the measured period, the solution pH was changed from pH 2 to pH 13 or from pH 13 to pH 2 and repeated the loop for 5 times independently. The time-lapsed optical micrographs were taken in 0.4 s-interval and processed to video format in 15 frames per second (fps). The scale bar is 20 µm.

**Title: Supplementary Movie 2.** Microscopic observation of tethered single sunflower pollen grain in aqueous solution. Over the measured period, the solution was changed from pH 7 to 10 mM KCl, then to 10 mM EDTA. The time-lapsed optical micrographs were taken in 0.4 s-interval and processed to video format in 15 frames per second (fps). The scale bar is 20 µm.

**Title: Supplementary Movie 3.** Microscopic observation of tethered single sunflower pollen grain in aqueous solution. Over the measured period, the solution was changed from pH 7 to 10 mM MgCl_2_, then to 10 mM EDTA. The time-lapsed optical micrographs were taken in 0.4 s-interval and processed to video format in 15 frames per second (fps). The scale bar is 20 µm.

**Title: Supplementary Movie 4.** Microscopic observation of tethered single sunflower pollen grain in aqueous solution. Over the measured period, the solution was changed from pH 7 to 10 mM CaCl_2_, then to 10 mM EDTA. The time-lapsed optical micrographs were taken in 0.4 s-interval and processed to video format in 15 frames per second (fps). The scale bar is 20 µm.

**Title: Supplementary Movie 5.** Microscopic observation of tethered single sunflower pollen grain in aqueous solution. Over the measured period, the solution was changed from pH 7 to 10 mM SrCl_2_, then to 10 mM EDTA. The time-lapsed optical micrographs were taken in 0.4 s-interval and processed to video format in 15 frames per second (fps). The scale bar is 20 µm.

**Title: Supplementary Movie 6.** Microscopic observation of tethered single sunflower pollen grain in aqueous solution. Over the measured period, the solution was changed from pH 7 to 10 mM Fe(NO_3_)_3_, then to 10 mM EDTA. The time-lapsed optical micrographs were 8 taken in 0.4 s-interval and processed to video format in 15 frames per second (fps). The scale bar is 20 µm.

**Title: Supplementary Movie 7.** Computational simulations when $M_{E/I}=1.6$.

**Title: Supplementary Movie 8.** Computational simulations when $M_{E/I}=0.7$.

**Title: Supplementary Movie 9.** Microscopic observation of tethered pollen grains in aqueous solution. Over the measured period, the solution was changed from pH 13 to pH 2, or from pH 2 to pH 13. The pollen species belong to eudicots, which are the same as sunflower. The pollens include baccharis, camellia, gala, lotus, motherwort, and poppy. The time-lapsed optical micrographs were taken in 0.4 s-interval and processed to video format in 15 frames per second (fps). The scale bar is 20 µm.
